# Modeling Strain Hardening of Metallic Materials with Sigmoidal Function Considering Temperature and Strain Rate Effects

**DOI:** 10.3390/ma17163950

**Published:** 2024-08-08

**Authors:** Boyu Pan, Fuhui Shen, Sanjay Raghav Sampathkumar, Sebastian Münstermann

**Affiliations:** Institute of Metal Forming, RWTH Aachen University, Intzestraße 10, 52072 Aachen, Germany; boyu.pan@ibf.rwth-aachen.de (B.P.); fuhui.shen@ibf.rwth-aachen.de (F.S.); sanjay.sampathkumar@ibf.rwth-aachen.de (S.R.S.)

**Keywords:** temperature, strain rate, hardening law, anisotropy, strength differential effect

## Abstract

This study uses a sigmoidal function to describe the plastic strain hardening of metallic materials, considering temperature and strain rate effects. The effectiveness of this approach is evaluated and systematically compared with other hardening laws. Incorporating temperature and strain rate effects into the parameters of this sigmoidal-type hardening law enables a more precise description and prediction of the plastic deformation of materials under different combinations of temperature and strain rate. The temperature effect is coupled using a simplified Arrhenius model, and the strain rate effect is coupled with a modified Johnson–Cook model. The sigmoidal-type hardening law is integrated with an asymmetric yield criterion to address complex behavior, such as anisotropy and strength differential effects. The calibration and validation of the constitutive model involve examining uniaxial tensile/compressive flow curves in various directions and biaxial tensile/compressive flow curves for diverse metallic alloys, proving the proposed model’s broad applicability.

## 1. Introduction

The accurate characterization of plastic deformation in metallic materials is crucial for research and industrial production, as inaccuracies can lead to poor predictions and design flaws [1,2,3,4]. An effective, easily implemented, and widely applicable model for flow behavior is essential because of this. Traditional hardening laws, like the Swift hardening law, Ludwik hardening law, and Voce hardening law, are suitable for materials with convex strain hardening behavior, such as body center cubic (BCC) and face center cubic (FCC) materials. However, they fall short for materials with complex behaviors, such as concave or sigmoidal-shape curves seen in certain hexagonal closed-packed (HCP) materials under tension loading. The inadequacy of traditional hardening laws is due to the complex transition in deformation mechanisms, such as twinning-dominant or slip-dominant behaviors in HCP materials [5,6,7]. Models by Muhammad et al. [8] and Nguyen et al. [9] address this issue but are complex, and the calibration process for the model parameters is excessively time-intensive, discouraging their broad implementation.

Additionally, many existing phenomenological models aiming at characterizing the sigmoidal hardening behavior of materials fail to validate their applicability and reliability beyond experimental ranges, such as the ones proposed by Kurukuri et al. [10], Clyne and Gu [11], and Mukarati et al. [12]. These models, while valid within controlled experimental parameters, struggle to maintain accuracy and predictive ability under more variable conditions. This limitation highlights the need for more robust models that can reliably extend predictions to cover varied material behaviors and environmental conditions.

Besides the non-convex shape, another distinct feature in the flow curves of HCP materials is the tension–compression asymmetry (TCA). This is also called the strength differential (SD) effect, which occurs due to the activation of twinning and different sensitivity to the preexisting flaws in materials [13,14,15]. Models proposed by Spitzig et al. [16], Cazacu and Barlat [17], Cazacu et al. [18], Gao et al. [19], Yoon et al. [20], and Hu and Yoon [21] have been applied to capture the flow behavior of materials that exhibit TCA. Their models incorporate either the first stress invariant $I_{1}$, or the effect of the third stress invariant $J_{3}$, or both $I_{1}$ and $J_{3}$, into the von Mises yield criterion so the asymmetric yielding can be modeled. The flexibility of these models can be improved by using linear transformation or summation methods, as presented in the work of Plunkett et al. [22] and Yoshida et al. [23]. In this way, the applications of these models are further extended by upscaling from isotropic materials to anisotropic cases.

Though success has been achieved, the models mentioned above are mainly focusing on applications at room temperature and quasi-static conditions. However, temperature and strain rate effects significantly impact plastic deformation and must be considered. Different approaches can be applied to integrate these effects into the plasticity model to coherently combine strain evolution, temperature influence, and strain rate effect. The first method involves directly including a hardening law into the model, such as the Johnson–Cook (JC) model [24], Bonder and Partom (BP) model [25], Zerilli and Armstrong (ZA) model [26], and the Khan–Huang–Liang (KHL) model [27,28]. These models either consider temperature and strain rate as independent factors, coupling them into the plastic deformation using a multiplicative method, or as interdependent factors, coupling them into the plastic deformation in a combined multiplication–addition approach. Success is achieved when the aforementioned models are applied to different materials. However, deviations may be induced when applying this approach since the temperature and strain rate effect-related parameters may not be constant but evolve with increasing plastic deformation. The other way is directly calibrating the temperature/strain rate effect parameters at each strain level to predict the corresponding stress. By connecting the stresses at different strains, a new flow curve with high accuracy can be generated since the temperature/strain rate effect is well captured as plastic deformation increases. This method was proposed and successfully validated by Shen et al. [29]. However, the calibration work can be enormously substantial when considering an extensive plastic strain range since many strain levels must be selected to ensure high accuracy.

In addition to models proposed from a macroscopic perspective, there are also microstructure-based constitutive models for plastic deformation, such as those proposed by Sedláček and Blum [30], Choi et al. [31], Páez et al. [32], and Han et al. [33]. They concentrated on the microstructural aspects influencing plastic deformation, offering valuable insights into how these factors can be integrated into macroscopic models. However, challenges can arise due to the application of microscopic measurement methods to detect microstructure evolution, especially at high temperature and strain rate conditions.

Overall, the advancements mentioned above highlight the continuing development in the field of the constitutive modeling of plasticity, particularly under complex loading conditions and in materials exhibiting unusual responses to external loadings. Researchers are working to develop models that not only fit experimental data but also provide accurate predictions for untested conditions. A general model that can characterize various flow behaviors under different complex loading conditions while being easy to calibrate and implement is highly desired in both academic research and industry.

To characterize the flow behavior of metallic materials under complicated loading situations, an evolutionary thermomechanical plasticity model was developed in this study. This study extends the application of the sigmoidal-type hardening law proposed by the Muhammad et al. [8] model, initially for the reverse compression portion of the cyclic curves, to more general loading conditions. Its reliability and applicability were validated by comparing the prediction results using other hardening laws and texture simulation results at extended strain. A parametric study of the influence of model parameters on the flow curve was also carried out. Section 3 confirms the model’s applicability across different materials and loading conditions. Section 4 presents the coupling of temperature and strain rate effects into the sigmoidal-type hardening law, proposing a novel method to derive flow curves under complex loading. The SD effect for materials with TCA is also considered.

## 2. A Sigmoidal-Type Hardening Law

Numerous hardening laws, including the Swift hardening law, Voce hardening law, and Ludwik hardening law, have been widely used to characterize the flow behaviors of BCC, FCC, and some HCP materials under tension, whose flow curves exhibit a convex shape. However, these hardening laws fail to accurately capture the flow curves with concave or sigmoidal shapes, such as some HCP materials under compression. This unusual response of materials to external loading originates from complex deformation mechanisms such as twinning and slip dominance [5,6,7]. Several studies, including those by Muhammad et al. [8], Nguyen et al. [9], Kurukuri et al. [10], Clyne and Gu [11], and Mukarati et al. [12], have proposed new hardening models to describe more complex flow behaviors and fit flow curves with different shapes under various loading conditions. Despite the advancements, these models introduce a large number of parameters, making the calibration process cumbersome, and they lack validation beyond the experimental strain range, thereby limiting the applicability and reliability.

In this study, Muhammad et al.’s [8] model for the reverse compression portion of the cyclic curves is extended to describe more general strain hardening behavior in various metallic materials under different loading conditions. The adapted model is given as

(1) $$\sigma_{T,0}(\varepsilon_{p})=\frac{F_{1}-F_{4}}{1+exp(-\left(\varepsilon_{p}-F_{2}\right)/F_{3})}+F_{4}$$

$F_{1}\sim F_{4}$ are four parameters that control the yield strength, strain hardening, and ultimate flow strength. They need to be calibrated based on experimental flow curves. In this study, the flow curve data, specifically the true stress and strain data, were input into MATLAB R2021b. The curve fitting tool in MATLAB was used. After giving the adapted model, the trust-region algorithm within the curve fitting tool was applied with a tolerance set to $10^{-8}$. By adjusting the starting points for $F_{1}\sim F_{4}$ based on the yield point of each flow curve, an optimal set of $F_{1}\sim F_{4}$ could be generated.

To validate the reasonableness of the sigmoidal hardening law, the work of Agnew et al. [34] was referred to for the following reasons:

(1) AZ31B was loaded by in-plane compression (IPC) and though-thickness compression (TTC) in the work of Agnew et al. [34], and the flow curve at TTC has a convex shape, while the flow curve at IPC has a sigmoidal shape;
(2) Agnew et al. [34] successfully used a texture simulation method to fit the experimental data within the testing strain range (for TTC, the experimental strain range was 0 to 0.4; for IPC, the experimental strain range was 0 to 0.275). The texture simulation method also managed to predict the flow behavior beyond the testing strain range (for TTC, the predicting strain range was 0 to 0.5; for IPC, the experimental strain range was 0 to 0.5). This provides a reliable basis for model evaluation.

All the experimental data and simulated data of AZ31B at TTC and IPC in the work of Agnew et al. [34] were obtained by graph reading and calculation, listed in Appendix A. Besides the sigmoidal-type hardening law, the experimental data were also fitted using the Swift, Ludwik, and Voce hardening laws for comparison. The fitted parameters for various materials with different hardening laws are listed in Table 1. The R-squared values are also listed in the table for straightforward comparison.

**Figure 1:**
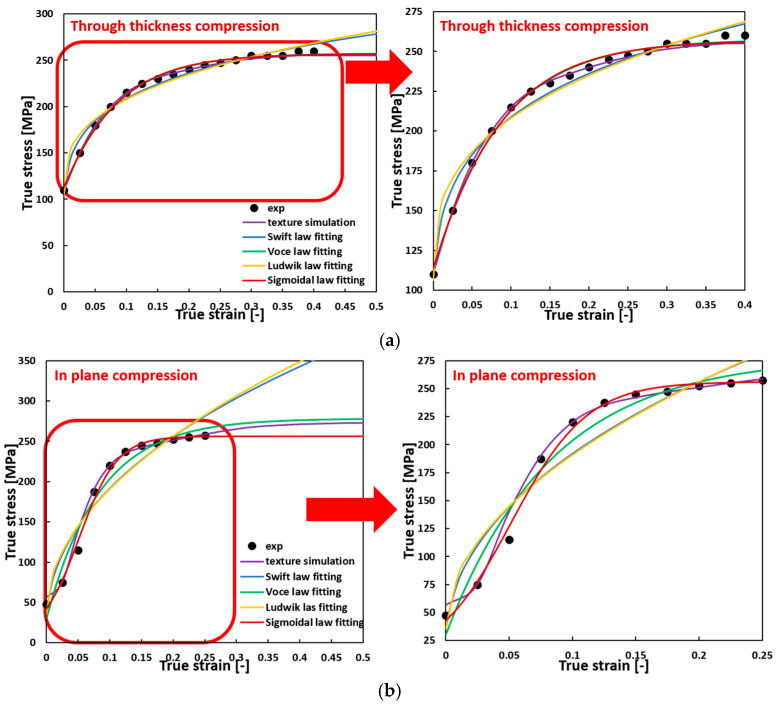
The flow curves described using different hardening laws compared to the experimental data and the texture-based simulation results for AZ31B under (**a**) TTC and (**b**) IPC.

With the fitted model parameters listed in Table 1, the flow curves can be drawn and compared with the experimental data and the simulated data provided by Agnew et al. [34] for AZ31B, as shown in Figure 1. Enlarged sections highlight the experimental strain ranges for better comparison, as indicated by a red arrow pointing from left to right in the graphs. For TTC with convex flow curves, both the flow curves fitted with the Voce hardening law and the sigmoidal-type hardening law aligned well with the experimental data and texture simulations, while the flow curves fitted with Swift and Ludwik’s laws do not fit accurately. Beyond the tested strain ranges, the flow curves fitted with the Voce and the sigmoidal laws closely match texture simulation results. For in-plane compression (IPC) with sigmoidal flow curves, only the flow curve fitted with the sigmoidal-type law captures the correct shape, while the flow curves fitted with other hardening laws show convex shapes. At extended strains, Swift and Ludwik’s laws overestimate flow stress, whereas the predictions by the Voce and the sigmoidal laws remain accurate. Based on these comparisons, the sigmoidal law’s superior performance for fitting both convex and sigmoidal flow curves and its reliable stress predictions at strains beyond testing ranges are highlighted.

**Figure 2:**
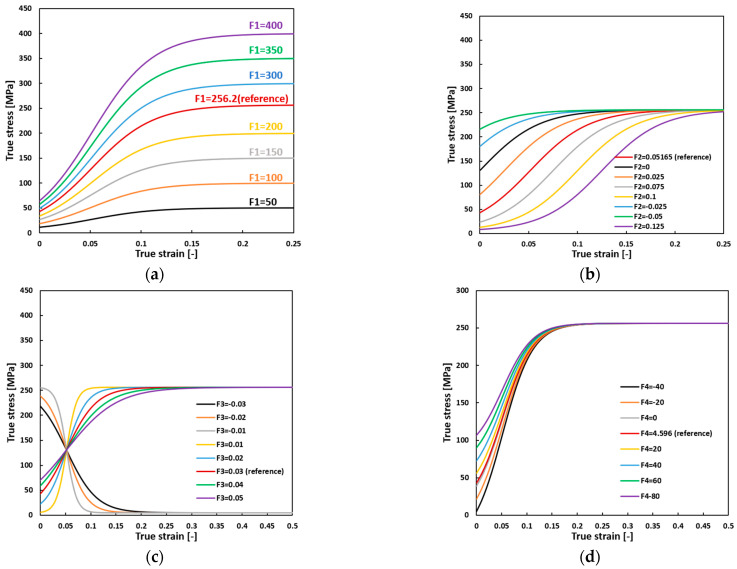
Parametric study on the influence of individual parameters of sigmoidal function on the flow curve (**a**) $F_{1}$, (**b**) $F_{2}$, (**c**) $F_{3}$, and (**d**) $F_{4}$.

Utilizing the parameters specified for IPC in Table 1, a parametric study is performed to evaluate the sensitivity of parameters $F_{1}-F_{4}$. The corresponding flow curves are shown in Figure 2. Notably, the entire flow curve experiences an upward shift, encompassing both yield strength and saturation flow stress, with an escalation in the strain hardening rate as the parameter $F_{1}$ increases. Changing parameter $F_{2}$ only affects the yielding point, lowering it and making the transition to ultimate strength sharper. Changing parameter $F_{3}$ singularly does not alter the ultimate strength but influences the yield strength and hardening rate. Modifying parameter $F_{4}$ alone does not affect the ultimate strength, but it raises the yielding point with an increasing $F_{4}$, thus contributing to a steeper transition from yielding to the ultimate strength. In summary, $F_{1}$ controls ultimate strength, while $F_{1}$, $F_{2}$, and $F_{4}$ control the yielding point and hardening rate. Positive or negative values of $F_{3}$ capture strain hardening and strain softening effects, respectively.

## 3. Application to BCC, FCC, and HCP Materials at Different Loading States

To further validate the application of the sigmoidal-type hardening law in various complicated loading situations, the experimental data of three types of materials were referred to and fitted using the sigmoidal-type hardening law. The three selected materials are isotropic Ta–10W (BCC) tested at different strain rates by Knezevic at el. [35], isotropic 2024-T351 aluminum (FCC) tested at various temperatures by Seidt and Gilat [36], and anisotropic AZ31 (HCP) loaded at various temperatures and different strain rates by Khan et al. [37]. All experimental data, as detailed in Appendix A, were acquired through graph reading and calculations. Remarkably, each of the chosen materials exhibits either slight or pronounced TCA. Analogous to the approach employed in the previous section, these experimental datasets were fitted using the Swift, Ludwik, and Voce hardening laws. The fitted flow curves were extrapolated to a strain of one for comparative analysis. The parameters derived from the fittings for various materials, using different hardening laws, are documented in Table A5, Table A6 and Table A7 in Appendix B. Additionally, R-squared values are included within the tables for straightforward comparisons.

**Figure 3:**
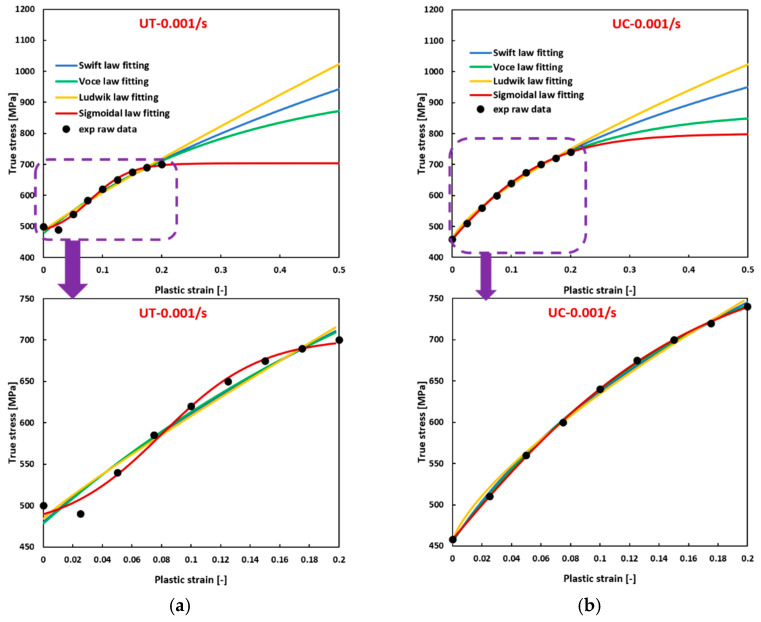

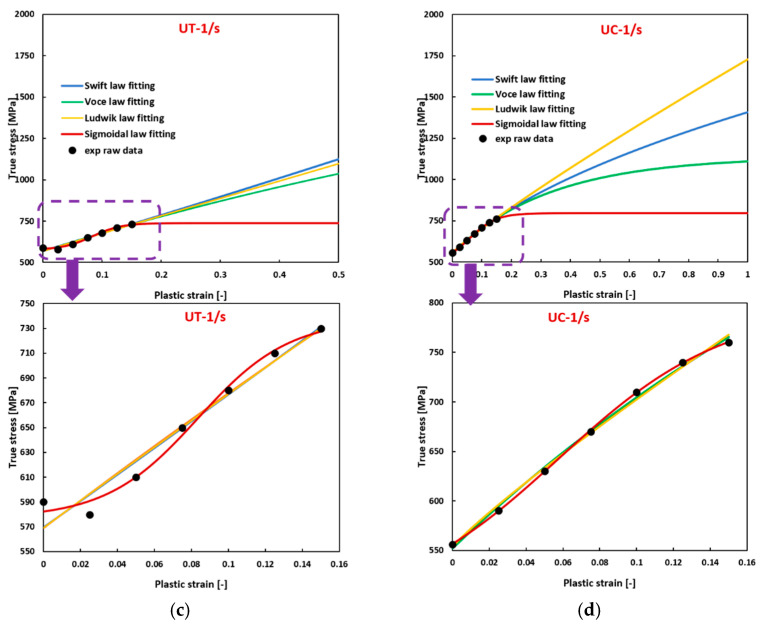
Comparison of the fitted flow curves and the experimental data for Ta-10W (BCC) using different hardening laws when (**a**) UT at 0.001/s; (**b**) UT at 1/s; (**c**) UC at 0.001/s; (**d**) UC at 1/s.

**Figure 4:**
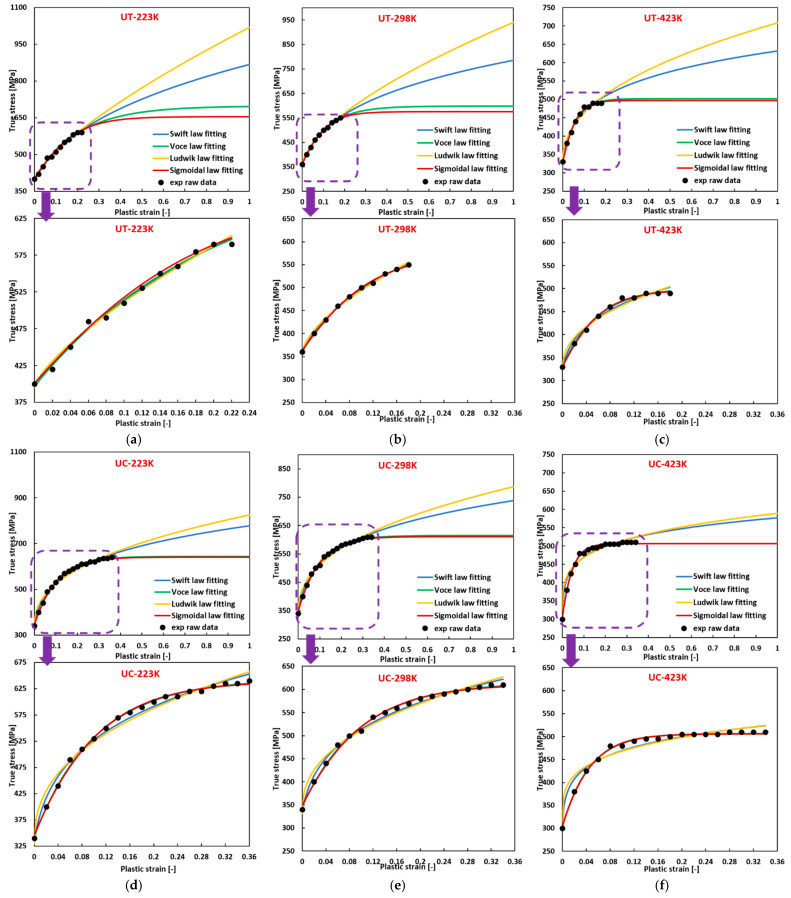
Comparison of the fitted flow curves and the experimental data for 2024-T351 (FCC) using different hardening laws when (**a**) UT at 223 K; (**b**) UT at 298 K; (**c**) UT at 423 K; (**d**) UC at 223 K; (**e**) UC at 298 K; (**f**) UC at 423 K.

**Figure 5:**
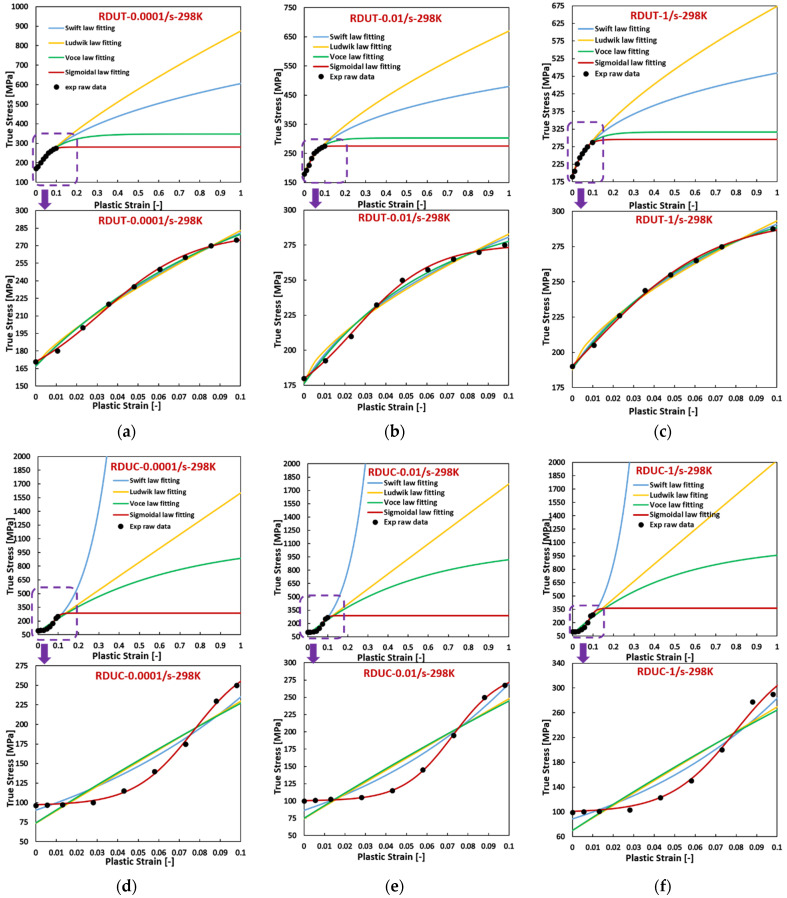

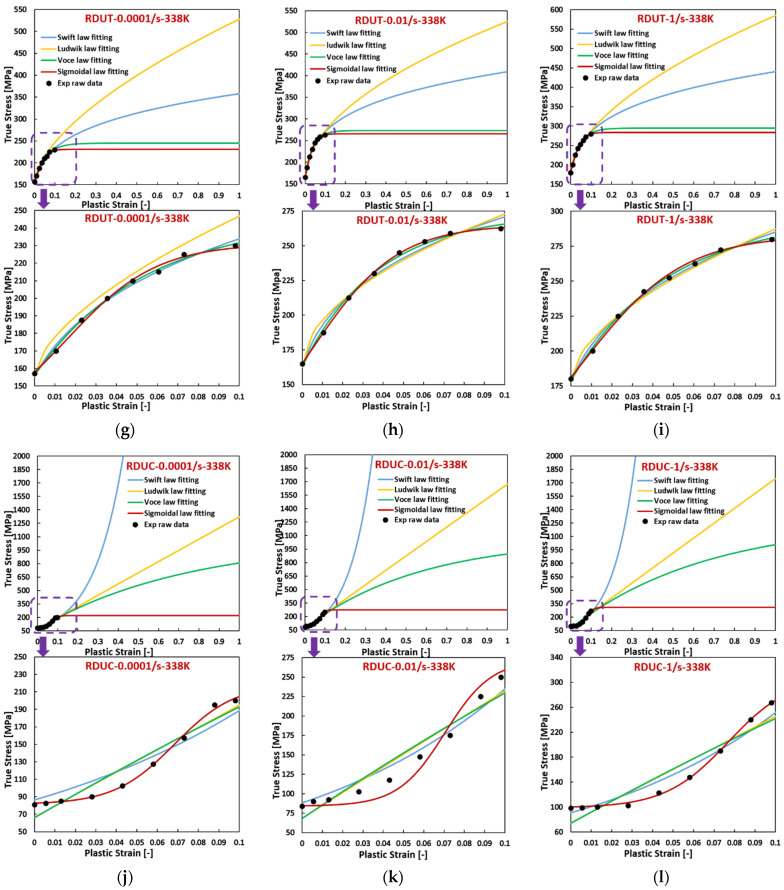

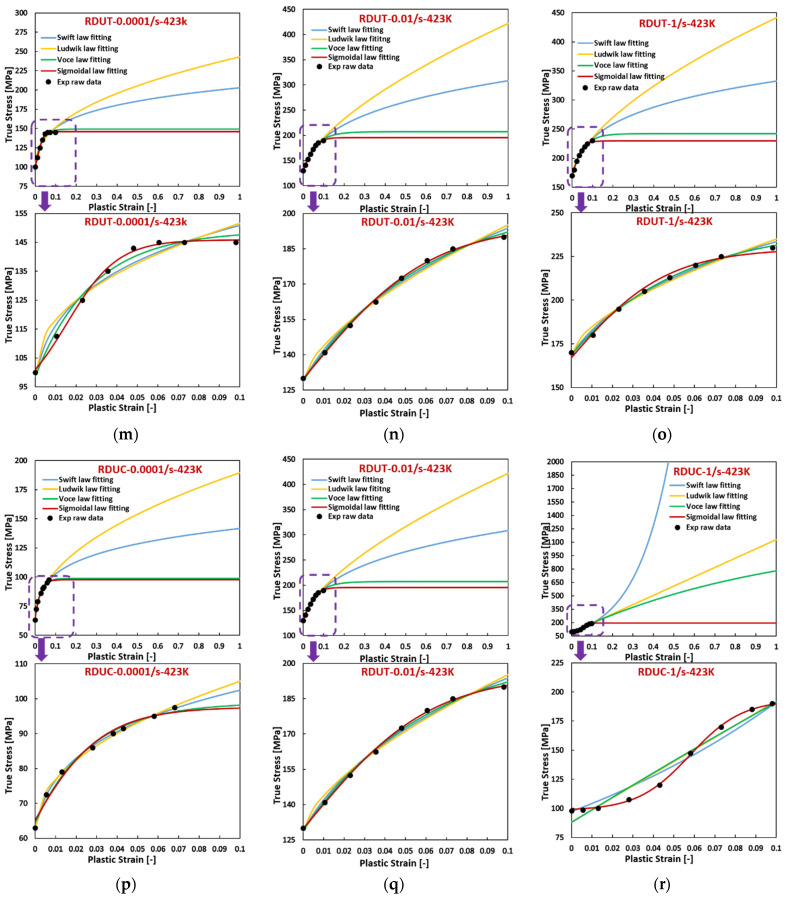
Comparison of the fitted flow curves and the experimental data for AZ31 (HCP) using different hardening laws at different loading states along RD. (**a**–**c**), loaded along UT at 298 K, the strain rate increases from 0.0001/s to 1/s. (**d**–**f**), loaded along UC at 298 K, the strain rate increases from 0.0001/s to 1/s. (**g**–**i**), loaded along UT at 338 K, the strain rate from 0.0001/s to 1/s. (**j**–**l**), loaded along UC at 338 K, the strain rate increases from 0.0001/s to 1/s. (**m**–**o**), loaded along UT at 423 K, the strain rate increases from 0.0001/s to 1/s. (**p**–**r**), loaded along UC at 423 K, the strain rate increases from 0.0001/s to 1/s.

**Figure 6:**
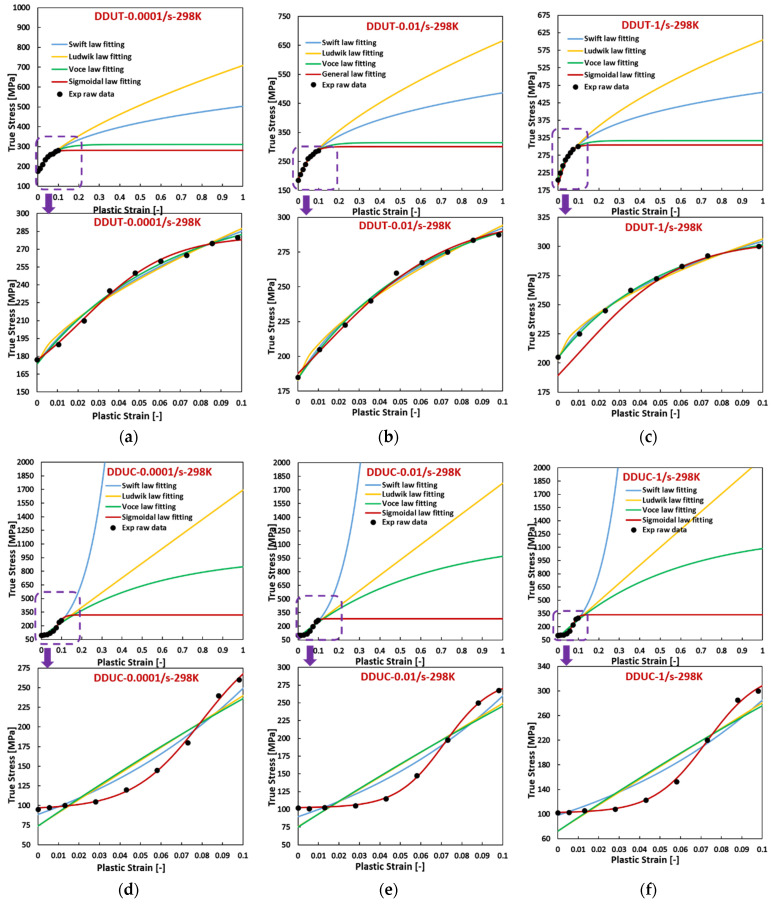

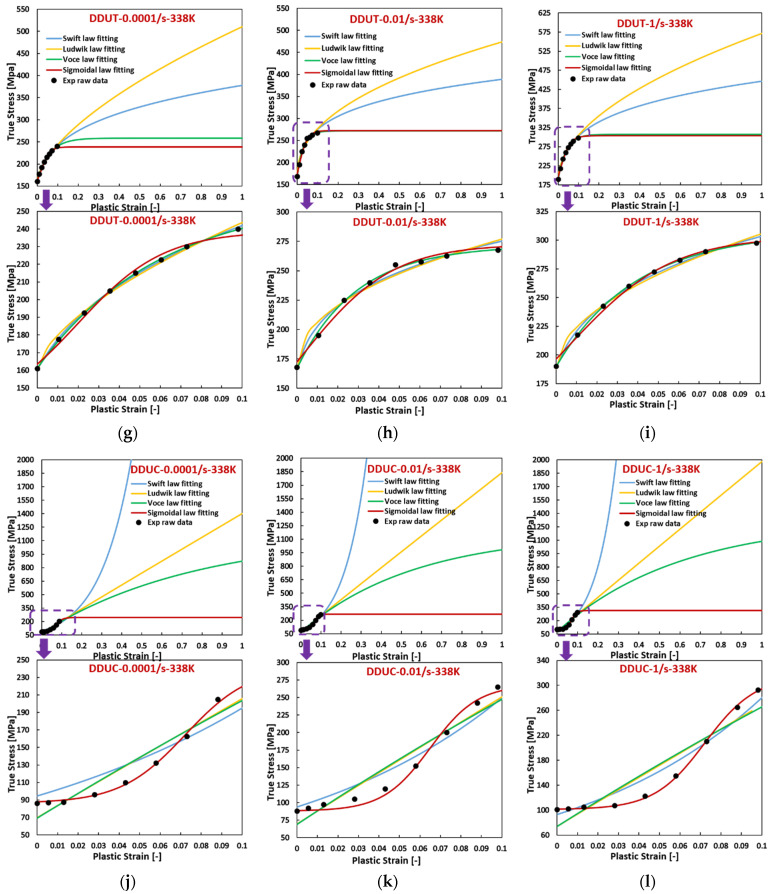

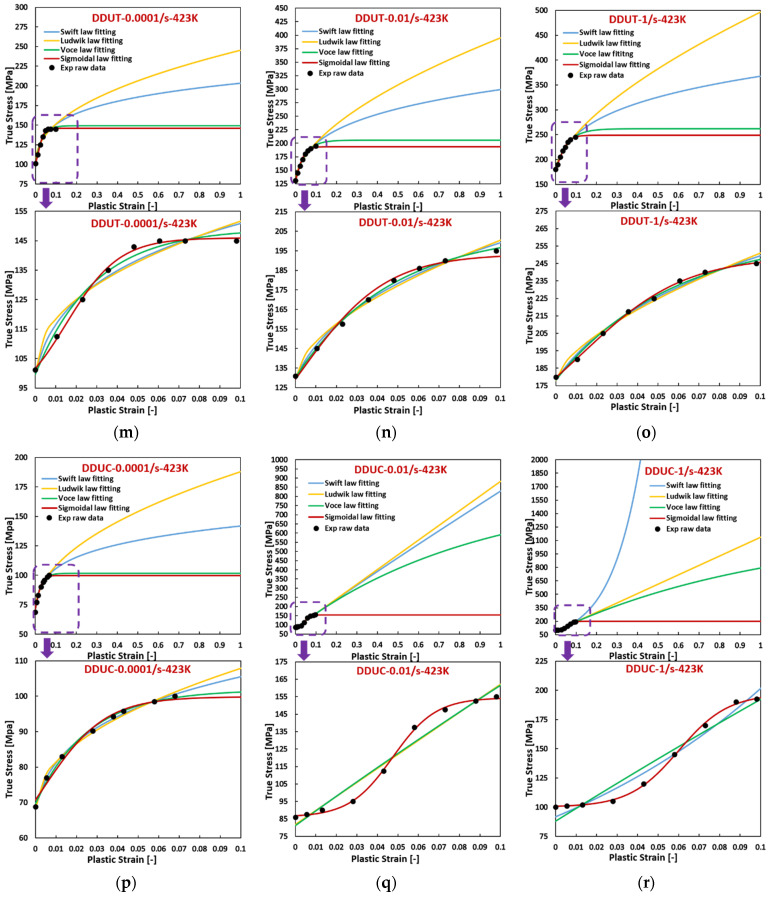
Comparison of the fitted flow curves and the experimental data for AZ31 (HCP) using different hardening laws at different loading states along DD. (**a**–**c**), loaded along UT at 298 K, the strain rate increases from 0.0001/s to 1/s. (**d**–**f**), loaded along UC at 298 K, the strain rate increases from 0.0001/s to 1/s. (**g**–**i**), loaded along UT at 338 K, the strain rate from 0.0001/s to 1/s. (**j**–**l**), loaded along UC at 338 K, the strain rate increases from 0.0001/s to 1/s. (**m**–**o**), loaded along UT at 423 K, the strain rate increases from 0.0001/s to 1/s. (**p**–**r**), loaded along UC at 423 K, the strain rate increases from 0.0001/s to 1/s.

**Figure 7:**
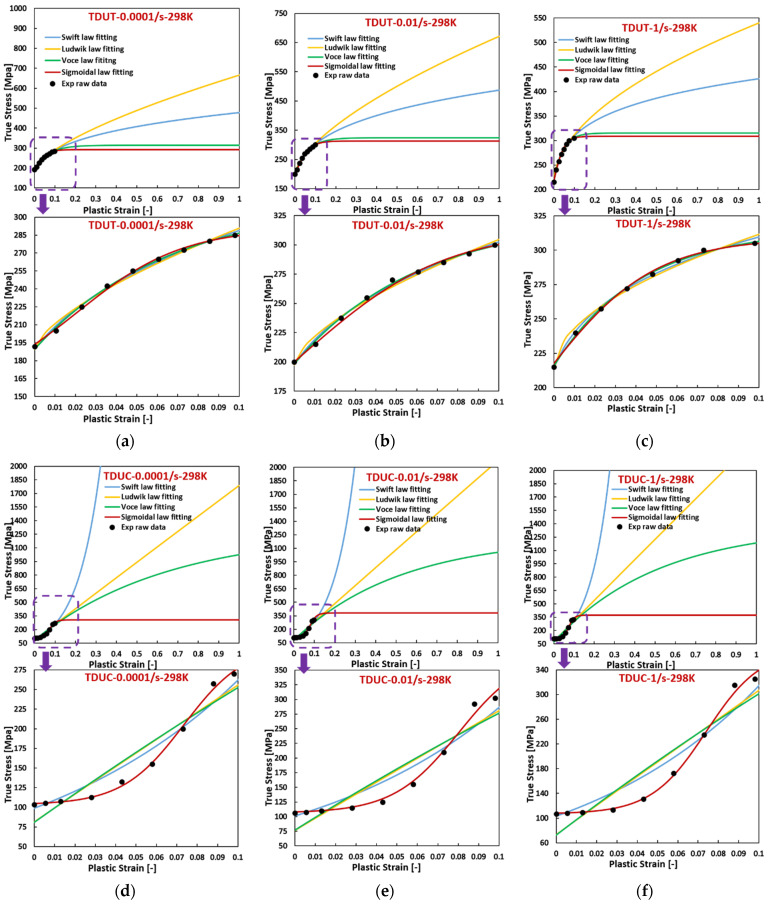

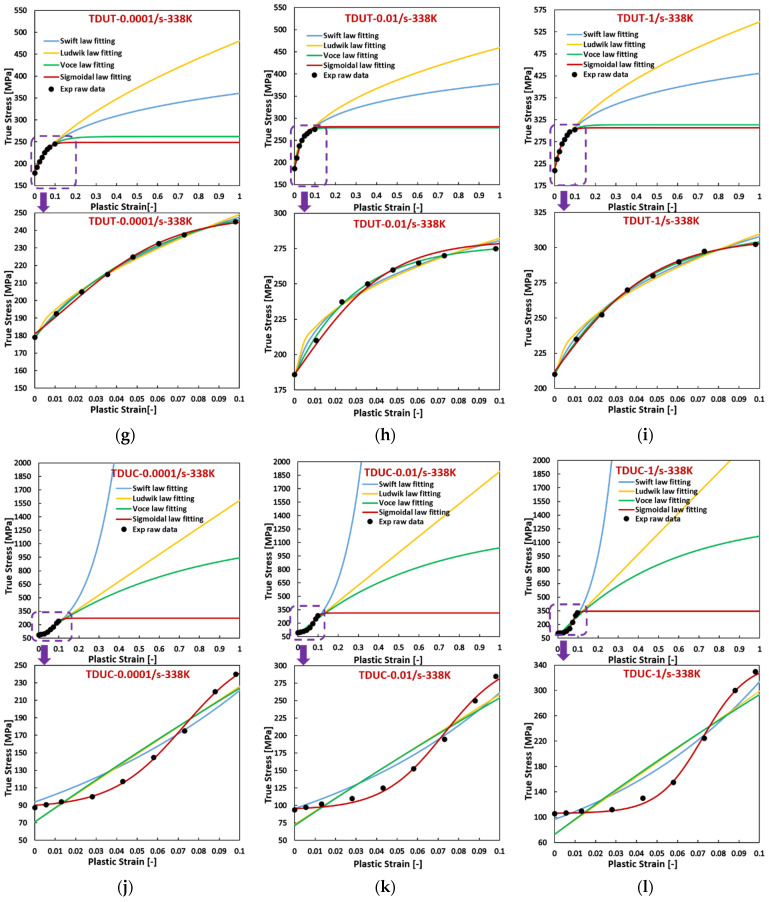

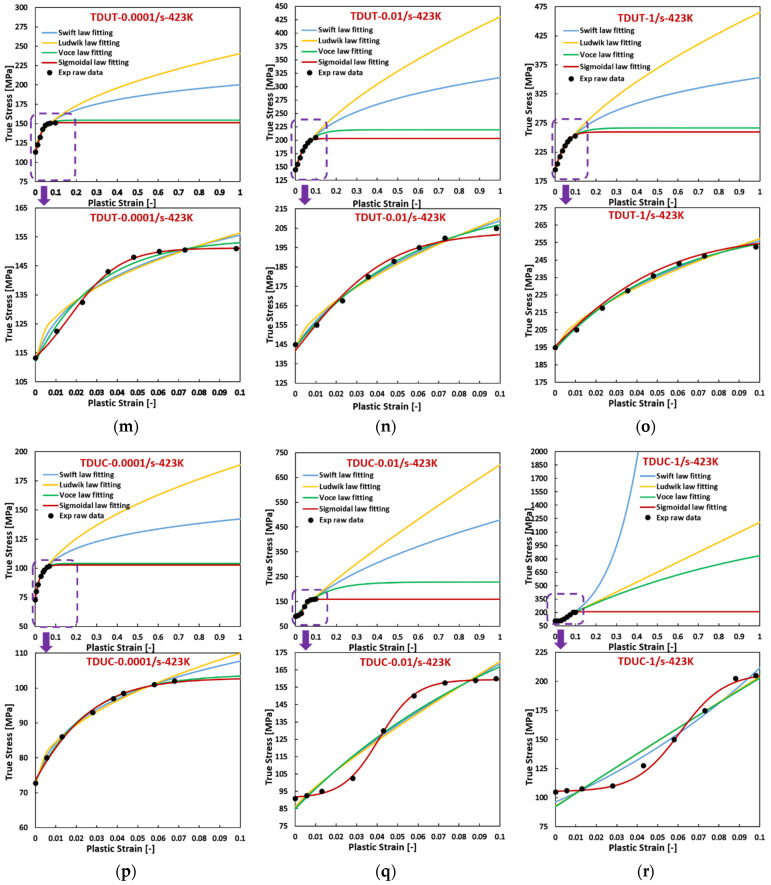
Comparison of the fitted flow curves and the experimental data for AZ31 (HCP) using different hardening laws at different loading states along TD. (**a**–**c**), loaded along UT at 298 K, the strain rate increases from 0.0001/s to 1/s. (**d**–**f**), loaded along UC at 298 K, the strain rate increases from 0.0001/s to 1/s. (**g**–**i**), loaded along UT at 338 K, the strain rate from 0.0001/s to 1/s. (**j**–**l**), loaded along UC at 338 K, the strain rate increases from 0.0001/s to 1/s. (**m**–**o**), loaded along UT at 423 K, the strain rate increases from 0.0001/s to 1/s. (**p**–**r**), loaded along UC at 423 K, the strain rate increases from 0.0001/s to 1/s.

Utilizing the model parameters in Table A5, Table A6 and Table A7, flow curves were constructed and compared with experimental data for the three specified materials, as depicted in Figure 3, Figure 4, Figure 5, Figure 6 and Figure 7. Enlarged figures are shown for the experimental strain ranges for better comparison, as indicated by a purple arrow pointing up to down in the graphs. Figure 3 demonstrates that, for Ta–10W (BCC) subjected to varying strain rates, its flow curves exhibit a convex shape only when compressed at 0.001/s, and for the rest of the conditions, they exhibit a sigmoidal shape, either severe or slight. The sigmoidal-type hardening law exhibits superior accuracy in fitting the experimental raw data, successfully preserving both the sigmoidal and convex shape of the flow curves under different conditions, while using other hardening laws can lead to big deviations, especially when the strain is extended beyond the experimental range. The flow curves fitted with the Ludwik hardening law show the most significant deviations. The flow curves fitted with the Voce hardening law are closest to those fitted with the sigmoidal-type hardening law but still deviate considerably from the experimental data. The R-squared values listed in Table A5 in Appendix B also demonstrate that using the sigmoidal-type hardening law can achieve the highest accuracy when fitting the flow curves at different strain rates. What’s more, compared with the sigmoidal-type hardening law, the others fail to capture the sigmoidal shape. In these figures, UT presents uniaxial tension, and UC represents uniaxial compression.

A similar trend is evident in Figure 4. For 2024-T351 aluminum (FCC) tested at varying temperatures, its flow curves exhibit convex shapes at all loading conditions. Within the experimental strain ranges, the fitted flow curves using different hardening laws show no significant difference in tension. The flow curves fitted with the Voce hardening law are closest to those fitted with the sigmoidal-type hardening law. The flow curves fitted with the Ludwik hardening law deviate the most from those fitted with the sigmoidal-type hardening law. However, notable differences can be observed when fitting the curves in compression, where the sigmoidal-type hardening law best matches the experimental data points. The precision of fitting using different hardening laws is further highlighted by examining the respective R-squared values, as presented in Table A6 in Appendix B, showing that the sigmoidal-type hardening law is the best choice for flow curve fitting for this material.

Figure 5, Figure 6 and Figure 7 reveal that, for AZ31 (HCP) tested under various temperatures and strain rates, flow curves fitted with all hardening laws accurately match experimental data when the experimental flow curves exhibit convex shapes, such as during tensile loading across all directions, temperatures, and strain rates, and compressive loading at 423 K with a strain rate of 0.0001/s. Particularly noteworthy is the ideal fit achieved by the sigmoidal-type hardening law for flow curves exhibiting pronounced sigmoidal shapes during compressive conditions at specific situations, such as 298 K with a strain rate of 0.01/s, 423 K with a strain rate of 0.01/s, and 423 K with a strain rate of 1/s. Conversely, under certain compressive conditions, such as 298 K with a strain rate of 0.0001/s, 338 K with a strain rate of 0.0001/s, 298 K with a strain rate of 1/s, and 338 K with a strain rate of 1/s, where the flow curves display slight sigmoidal shapes, the sigmoidal-type hardening law exhibits superior fitting compared to the other hardening laws. However, under specific compressive conditions, such as 423 K with a strain rate of 0.0001/s and 338 K with a strain rate of 0.01/s, where the flow curves manifest convex shapes, the sigmoidal-type hardening law does not demonstrate a distinctive advantage over other hardening laws. In these graphs, RD, DD, and TD represent the rolling direction, diagonal direction, and transverse direction, respectively. As an example, RDUT means uniaxial tension along the rolling direction. The sigmoidal-type hardening law is superior in both fitting accuracy and applicability for characterizing flow curves exhibiting either convex or sigmoidal shapes within the experimental testing ranges. Furthermore, when strain is extrapolated to values beyond those obtained experimentally, such as 50% or 100%, the fitting curve extrapolated using the sigmoidal-type hardening law exhibits no singularity point. Its growth pattern aligns closely with that of the Voce hardening law, though slightly lower, particularly when the flow curve exhibits a convex shape, as illustrated in Figure 1.

This observation is substantiated by the fact that, under conditions where the material’s flow curve shows a sigmoidal shape, the Swift, Voce, and Ludwik hardening laws fail to achieve accurate fitting and cannot characterize the sigmoidal shape effectively. In contrast, the sigmoidal-type hardening law precisely characterizes the sigmoidal shape of the flow curve and generates reasonable stress values, particularly when the plastic strain reaches significant magnitudes. This assertion is directly validated by comparing flow curves fitted using different hardening laws against the texture simulation results, as depicted in Figure 1.

In summary, the sigmoidal-type strain hardening law, initially adopted by Muhammad et al. [8] for the reverse compression portion of the cyclic curves, has been successfully extended to describe the strain hardening properties of various metallic materials under more general loading conditions. For different BCC, FCC, and HCP materials with either convex or concave shape flow curves, the applicability of the sigmoidal-type hardening law under a large strain range is validated by comparing the predictions using this hardening law with the texture simulation results.

## 4. Coupling Temperature and Strain Rate Effect

### 4.1. General Methodology

Different from many other models that directly multiply the temperature effect, strain rate effect, and strain evolution into the stress, such as the Johnson–Cook (JC) model [24] and the modified JC model proposed by Shin and Kim [38], this study couples the temperature-related thermal softening effect and the strain rate effect into the strain evolution through the hardening law. This approach avoids deviations at high plastic strain by ensuring that temperature and strain rate effects change with increasing plastic strain, making calibration easier and faster.

By coupling the temperature and strain rate effect into the hardening law parameters, the model generates new flow curves at different temperature and strain rates, and the evolution of plastic strain is inherently considered. The involved parameters are fewer and easier to understand, thus making the calibration procedure relatively easy and fast.

**Figure 8:**
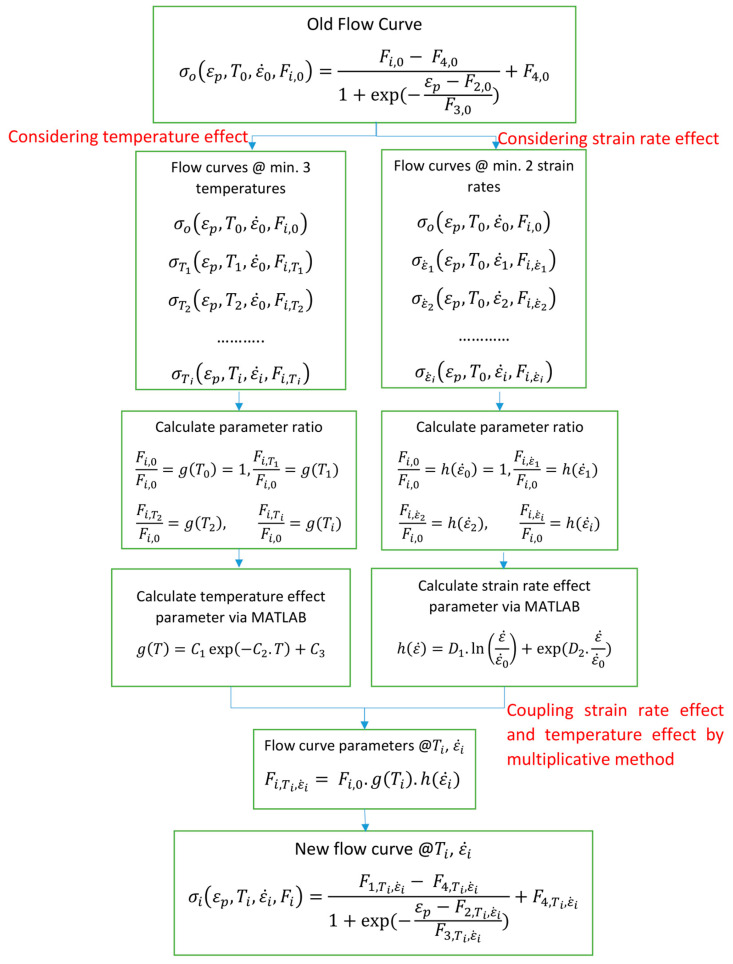
Flow chart of calibrating temperature and strain rate effect parameter and generating new flow curve according to Equation (2).

In this study, a simple Arrhenius-type function with three parameters ($C_{1-3}$) describing the thermal softening effects and a modified JC function proposed by Shin and Kim [38] with two parameters ($D_{1-2}$) addressing the strain rate effects are coupled into the material parameters of the sigmoidal-type hardening law. As a result, a new flow curve can be generated. The Arrhenius-type function explains the thermal softening physically by showing that the thermal activation energy of short-range barriers increases proportionally with temperature. The short-range barriers contribute significantly to the temperature-dependent components of flow stress. Incorporating the strain rate component from the modified JC model proposed by Shin and Kim [38] enhances the model’s flexibility and applicability by accurately capturing the rapid upturn in flow stress at high strain rates with an exponential dependence on the normalized strain rate. By coupling these two models, the material parameters can be exclusively determined by a constrained set of experimental results without introducing additional material properties, such as melting temperatures in the original JC model and KHL model. It simplifies the model formulation, implementation, and calibration and has been successfully validated by Pan et al. [39]. A general expression for the flow stress coupling temperature-related thermal softening effect, strain rate effect, and strain evolution is expressed as the following:

(2a) $$\sigma \left(\varepsilon_{p},T,\dot{\varepsilon }\right)=\frac{F_{1,T,\dot{\varepsilon }}-F_{4,T,\dot{\varepsilon }}}{1+exp(-\left(\varepsilon_{p}-F_{2,T,\dot{\varepsilon }}\right)/F_{3,T,\dot{\varepsilon }})}+F_{4,T,\dot{\varepsilon }}$$

(2b) $$F_{i,T,\dot{\varepsilon }}=F_{i,0}\cdot g(T)\cdot h(\dot{\varepsilon })$$

(2c) $$g(T)=C_{1}\cdot \exp\left(-C_{2}\cdot T\right)+C_{3}$$

(2d) $$h(\dot{\varepsilon })=D_{1}\cdot \ln\left(\frac{\dot{\varepsilon }}{{\dot{\varepsilon }}_{0}}\right)+exp(D_{2}\cdot \frac{\dot{\varepsilon }}{{\dot{\varepsilon }}_{0}})$$

where $F_{i}$ is the material parameter in the sigmoidal hardening law and i ranges from 1 to 4. $F_{i,0}$ is the parameter calibrated at the reference state, typically at room temperature and quasi-static loading. The parameter calibration procedure is shown in Figure 8. The temperature effect parameters $C_{1-3}$ and the strain rate effect parameters $D_{1-2}$ can be fitted with the curve fitting tool in MATLAB. When there is only the temperature or strain rate effect, g(T) or $h(\dot{\varepsilon })$ is set to one.

The analytical Yoon2014 model is adopted in this study and the final yield criterion is formulated as follows:

(3) $$f=\bar{\sigma }\left(a,b_{ij},c_{ij}|I_{1},J_{2}^{\prime },J_{3}^{\prime }\right)-\sigma \left(\varepsilon_{p},\;T,\;\dot{\varepsilon }\right)\leq 0$$

With this criterion, the yielding and plastic deformation behavior can be characterized and predicted with analytical solutions for BCC, FCC, and HCP materials (either isotropic or anisotropic) tested at different loading conditions. This assures the model’s broad applicability. The procedure for getting an analytical solution for Equation (3) can be referred to in the work of Hu and Yoon [21] and Pan et al. [39].

### 4.2. Modeling Strain Rate Effects in BCC Material Ta–10W

**Figure 9:**
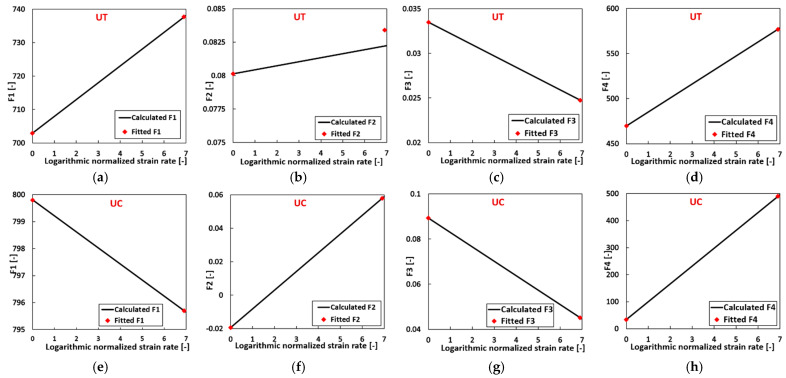
Comparison of the calculated and fitted material parameter $F_{i}$ in the sigmoidal-type hardening law at (**a**) $F_{1}$, (**b**) $F_{2}$, (**c**) $F_{3}$, (**d**) $F_{4}$ at UT; (**e**) $F_{1}$, (**f**) $F_{2}$, (**g**) $F_{3}$, (**h**) $F_{4}$ at UC.

Following the flow chart shown in Figure 8, and with the flow curve parameters of Ta–10W (BCC) at 0.001/s and 1/s listed in Table A5, the strain rate hardening parameters at tension and at compression can be calibrated separately based on Equation (2d), and they are listed in Table 2. The comparison of the calculated flow curve parameters and the fitting ones listed in Table A5 is shown in Figure 9.

**Figure 10:**
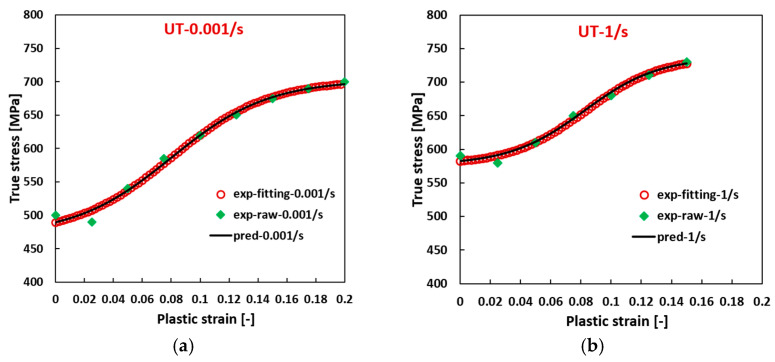

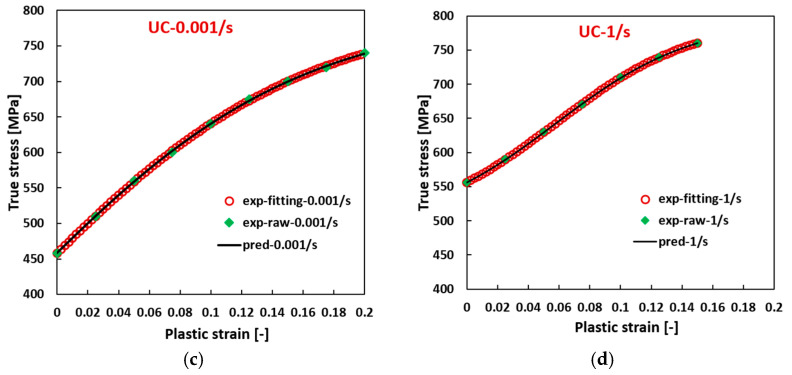
Comparison of the experimental stress, the fitting experimental flow curve, and the predicted flow curve for Ta–10W (BCC): (**a**) 0.001/s, (**b**) 1/s at UT, (**c**) 0.001/s, (**d**) 1/s at UC.

The computed material parameters $F_{i}$ match the fitted ones very well. With the calculated flow curve parameters at different strain rates, new flow curves can be generated at the corresponding strain rate. Figure 10 compares the fitted experimental flow curves generated with the material parameters listed in Table 2 and the predicted flow curves. The experimental data are also included inside. A perfect match can be observed among them. The formulated model effectively predicts $\sigma_{UT}$ and $\sigma_{UC}$ at elevated strain rates with substantial plastic deformation, providing a comprehensive representation of the general strain-rate-dependent evolution for Ta–10W (BCC).

### 4.3. Modeling Temperature Effects in FCC Material 2024-T351

**Figure 11:**
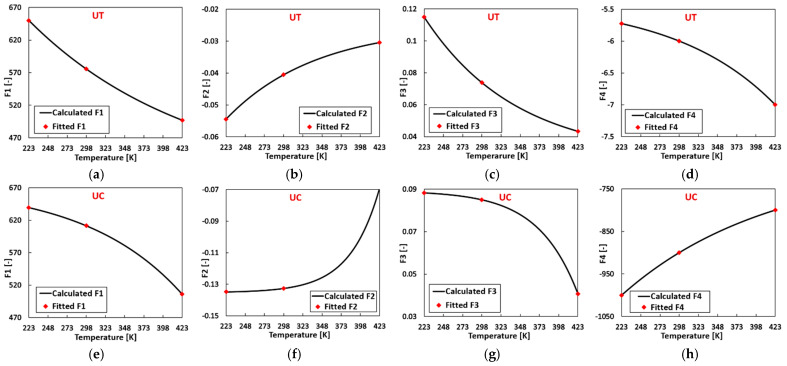
Comparison of the calculated and fitted material parameter $F_{i}$ in the sigmoidal-type hardening law: (**a**) $F_{1}$, (**b**) $F_{2}$, (**c**) $F_{3}$, (**d**) $F_{4}$ at UT; (**e**) $F_{1}$, (**f**) $F_{2}$, (**g**) $F_{3}$, (**h**) $F_{4}$ at UC.

Taking the steps illustrated in Figure 8 and utilizing the flow curve parameters for 2024-T351 (FCC) at temperatures of 223 K, 298 K, and 423 K listed in Table A6, the temperature-related thermal softening parameters for tension and compression can be independently calibrated using Equation (2c). The resulting parameters are presented in Table 3. Figure 11 compares the computed flow curve parameters and the fitted ones, revealing a close alignment between the calculated and fitted material parameters.

**Figure 12:**
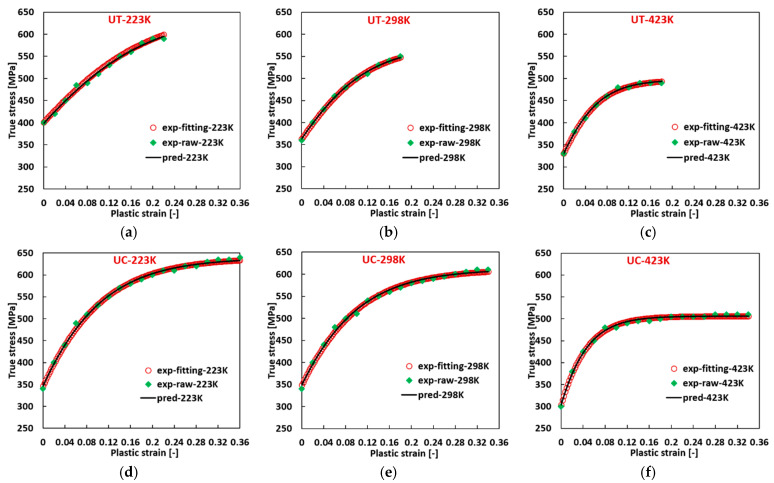
Comparison of the experimental stress, the fitted experimental flow curve, and the predicted flow curve for 2024-T351 (FCC) at quasi-static loading (**a**) 223 K, (**b**) 298 K, (**c**) 423 K at UT; (**d**) 223 K, (**e**) 298 K, (**f**) 423 K at UC.

Subsequently, with the derived flow curve parameters at varying temperatures, new flow curves can be constructed at the corresponding temperature using Equation (2a). The concordance between the fitted experimental flow curves generated with the material parameters listed in Table A6 and the predicted flow curves is illustrated in Figure 12, where the experimental data are also included. The predicted flow curves exhibit a commendable agreement with the fitted experimental flow curves and the experimental results.

### 4.4. Modeling Coupled Temperature and Strain Rate Effects in HCP Material AZ31

Following the flow chart shown in Figure 8, the temperature-related thermal softening parameters along all loading directions can be calibrated with the flow curve parameters at 298 K, 338 K, and 423 K with a strain rate of 0.0001/s based on Equation (2c), as listed in Table 4. Similarly, with the flow curve parameters at 0.0001/s, 0.01/s, and 1/s tested at 298 K along all loading directions, the strain rate hardening parameters along all loading directions can be calibrated based on Equation (2d), as listed in Table 5.

**Figure 13:**
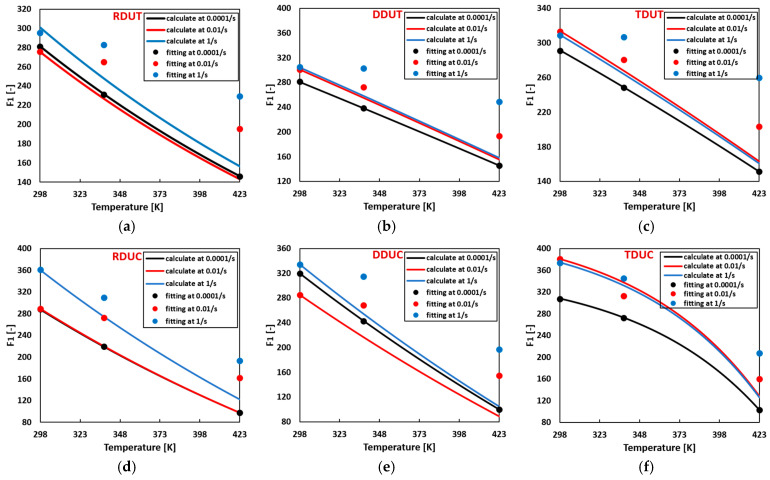
Comparison of the calculated and fitted material parameter $F_{1}$ in the sigmoidal-type hardening law at (**a**) RDUT, (**b**) DDUT, (**c**) TDUT, (**d**) RDUC, (**e**) DDUC, (**f**) TDUC.

**Figure 14:**
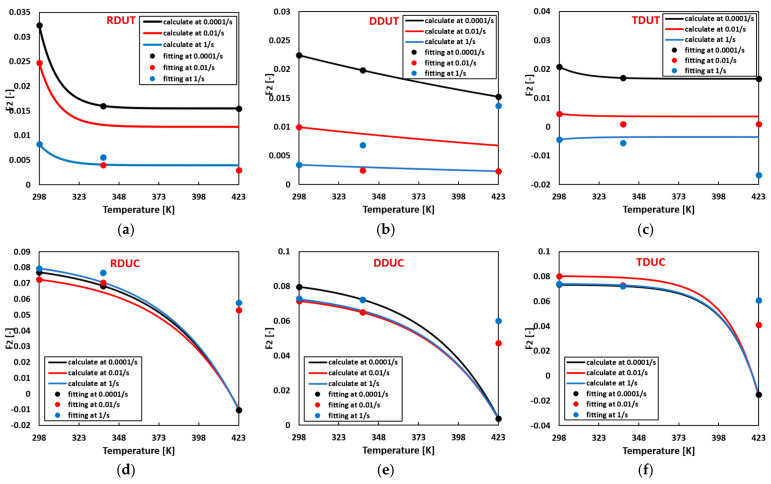
Comparison of the calculated and fitted material parameter $F_{2}$ in the sigmoidal-type hardening law at (**a**) RDUT, (**b**) DDUT, (**c**) TDUT, (**d**) RDUC, (**e**) DDUC, (**f**) TDUC.

**Figure 15:**
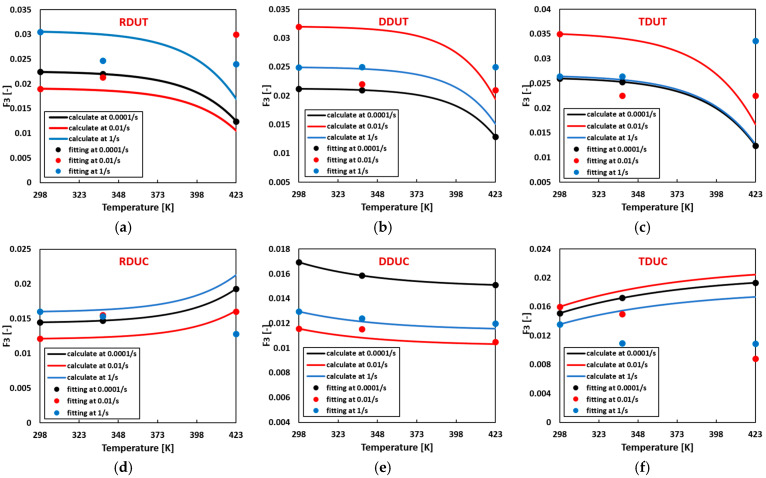
Comparison of the calculated and fitted material parameter $F_{3}$ in the sigmoidal-type hardening law at (**a**) RDUT, (**b**) DDUT, (**c**) TDUT, (**d**) RDUC, (**e**) DDUC, (**f**) TDUC.

**Figure 16:**
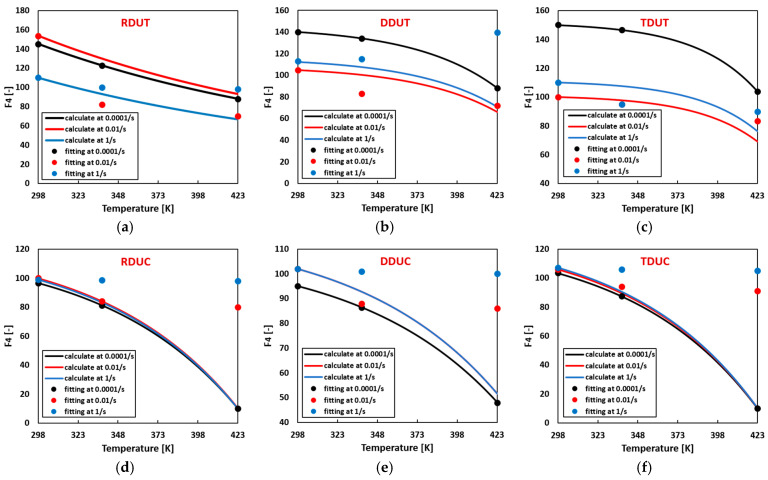
Comparison of the calculated and fitted material parameter $F_{4}$ in the sigmoidal-type hardening law at (**a**) RDUT, (**b**) DDUT, (**c**) TDUT, (**d**) RDUC, (**e**) DDUC, (**f**) TDUC.

Flow curve parameters can be computed for arbitrary temperature and strain rate combinations using the temperature-related thermal softening parameters summarized in Table 4 and the strain rate hardening parameters summarized in Table 5. Figure 13, Figure 14, Figure 15 and Figure 16 compare the calculated flow curve parameters and the fitting flow curves generated using the parameters listed in Table A7. The results indicate that when either the temperature or strain rate is individually increased, the calculated material parameters $F_{i}$ closely align with the fitted ones. However, deviations between the calculated and fitted material parameters gradually emerge when the temperature and strain rate are concurrently increased.

**Figure 17:**
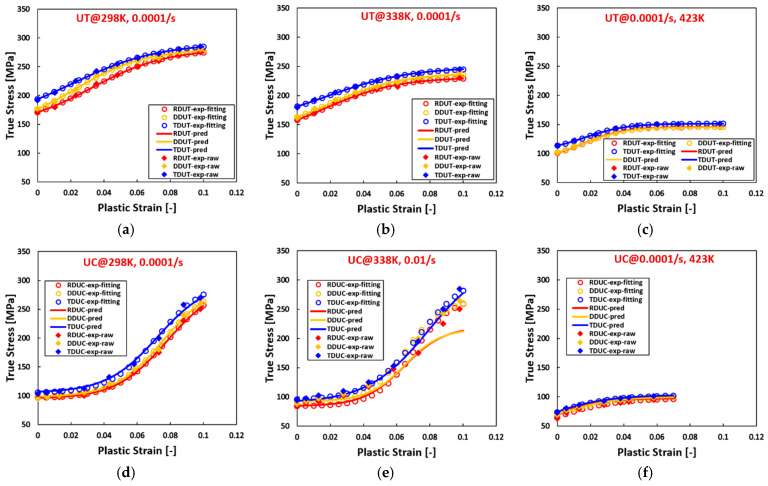
Comparison of the experimental stress, the fitted experimental flow curve, and the predicted flow curve at the strain rate of 0.0001/s: (**a**) 298 K, (**b**) 338 K, (**c**) 423 K at UT; (**d**) 298 K, (**e**) 338 K, (**f**) 423 K at UC.

**Figure 18:**
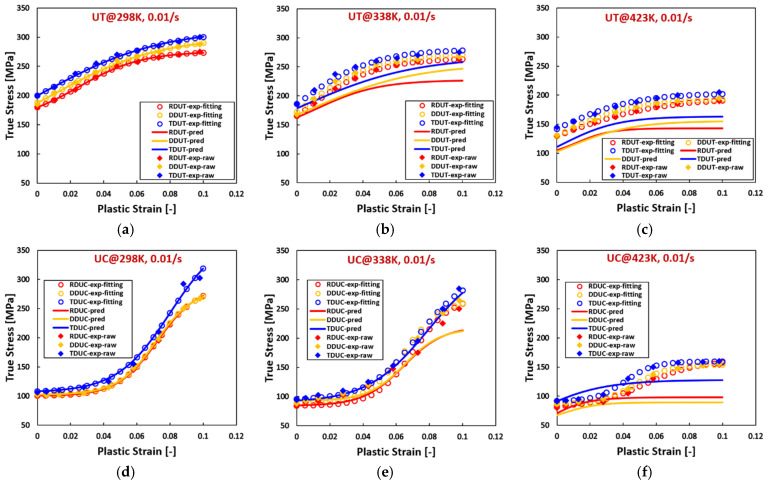
Comparison of the experimental stress, the fitted experimental flow curve, and the predicted flow curve at the strain rate of 0.01/s: (**a**) 298 K, (**b**) 338 K, (**c**) 423 K at UT; (**d**) 298 K, (**e**) 338 K, (**f**) 423 K at UC.

**Figure 19:**
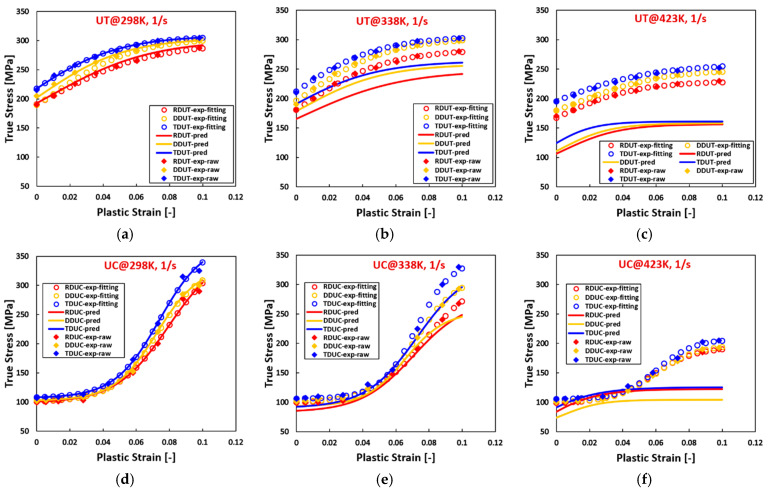
Comparison of the experimental stress, the fitted experimental flow curve, and the predicted flow curve at the strain rate of 1/s: (**a**) 298 K, (**b**) 338 K, (**c**) 423 K at UT; (**d**) 298 K, (**e**) 338 K, (**f**) 423 K at UC.

With the computed flow curve parameters for various temperatures and strain rates, new flow curves can be formulated. Figure 17, Figure 18 and Figure 19 compare the fitted experimental and the predicted flow curves, where the experimental data are also included. The sigmoidal-type hardening law successfully characterizes flow curves exhibiting convex and sigmoidal shapes. Particularly noteworthy is the successful prediction of sigmoidal compressive flow curves under various conditions. However, deviations between the predicted and fitted experimental flow curves and the experimental results become apparent when both the temperature and strain rate increase. The model encounters challenges in predicting the sigmoidal shape of the flow curve when the temperature is 423 K and the strain rate is 0.01/s and 1/s.

The deviations existing in both the prediction of the material parameter $F_{i}$ and flow curves can be explained as follows:

- The calibration of the temperature-related thermal softening parameters and strain rate effect parameters for material parameter $F_{i}$ is based on the flow curves of the following loading conditions: (a) temperature = 298 K, strain rate = 0.0001/s; (b) temperature = 298 K, strain rate = 0.01/s; (c) temperature = 298 K, strain rate = 1/s; (d) temperature = 338 K, strain rate = 0.0001/s; (e) temperature = 423 K, strain rate = 0.0001/s. The temperature and strain rate effects are integrated multiplicatively into the material parameter $F_{i}$, namely, the temperature-related thermal softening and strain rate effects are combined multiplicatively to predict stress flow stress. Consequently, at the aforementioned loading conditions, both the material parameter $F_{i}$ and flow curves are predicted with high accuracy, while deviations may arise under alternative loading conditions. This inherent limitation is intrinsic to the multiplicative method. Despite this limitation, the predictions using the proposed model generally align well with experimental data, handling complex scenarios involving temperature, strain rates, plastic deformation, anisotropy, and SD effects effectively.
- In the original work of Khan et al. [37], at a temperature of 423 K and a strain rate of 0.0001/s, all uniaxial compressive flow curves exhibit a convex shape, indicating saturating hardening behavior, with a maximum plastic strain of approximately 0.07. Under other conditions, the curves show a sigmoidal shape with higher plastic strains. This discrepancy, along with varied hardening behaviors, particularly at higher temperatures and strain rates, causes deviations. The observed alterations in hardening behavior with increasing strain rate during compression can be attributed to the activation of twinning modes with rising strain rates. Nevertheless, given the primary focus on model construction and validation in this study, a detailed exploration of the specific temperature and strain rate conditions leading to the occurrence of sigmoidal flow curves is not pursued in this discussion.

Generally speaking, the multiplicative method has limitations due to its inherent nature. Specifically, the effects of temperature and strain rate are coupled multiplicatively, and the involved parameters are calibrated individually. However, studies show that temperature affects the strain rate sensitivity [40,41,42]. This indicates that it is necessary to consider the interactive influence between the temperature and strain rate in some cases. What’s more, it is assumed in the current model that only thermal softening occurs with increasing temperatures, while there are studies indicating that the dynamic strain aging (DSA) effect should also be considered in some cases [43,44,45]. As a result, deviations can occur when predicting flow curves in significantly complex situations. Consequently, our model is suitable for materials where the effects of the temperature and strain rate do not significantly influence each other, and DSA does not occur. However, considering the noteworthy prediction accuracy for material parameters $F_{i}$ for flow curves, the temperature effect parameters $C_{1-3}$, and the strain rate effect parameters $D_{1-2}$, our model and method remain effective in predicting plastic strain hardening behavior under various complicated evolutionary conditions.

## 5. Summary and Conclusions

The applicability of the sigmoidal-type function in describing the strain hardening properties of various metallic materials, considering temperature and strain rate effects, has been systematically investigated and validated in this study. Key conclusions include:

- The sigmoidal-type hardening law accurately describes flow curves for materials with both convex and concave (or sigmoidal) shapes.
- Integrating a simple Arrhenius-type model for temperature effects and a modified Johnson–Cook model for strain rate effects into the parameters of the sigmoidal hardening law accurately describes material plastic flow stress under varying conditions.
- The influences of the temperature and strain rate on the strength differential effects and anisotropy are well-captured by integrating an advanced asymmetric yield criterion into the thermomechanical plasticity model, showing broad applicability for various metallic materials.
- Although the model is highly recommended for materials exhibiting both convex and concave (or sigmoidal) flow curves under various conditions, deviations may still exist due to the inherent limitation of the multiplicative approach. Future research should focus on introducing additional parameters to eliminate this limitation and increase model accuracy. The dynamic strain aging effect will also be considered in the future.

## Figures and Tables

**Table 1:** Fitted model parameters for AZ31B at TTC and IPC loading states.

Swift Hardening Law	$\sigma_{\varepsilon_{p},\theta ,T,\dot{\varepsilon }}=A*{\left(\varepsilon_{p}+B\right)}^{n}$	
Loading state	Parameter	
TTC	A	315.1
	B	0.002757
	n	0.181
	R-squared	0.9808
IPC	A	504.2
	B	0.002686
	n	0.4243
	R-squared	0.9169
**Ludwik hardening law**	$\sigma_{\varepsilon_{p},\theta ,T,\dot{\varepsilon }}=\sigma_{y}+k*{\varepsilon_{p}}^{n}$	
**Loading state**	**Parameter**	
TTC	$\sigma_{y}$	106.2
	k	221.3
	n	0.3372
	R-squared	0.9689
IPC	$\sigma_{y}$	35.81
	k	497.8
	n	0.5065
	R-squared	0.9094
**Voce hardening law**	$\sigma_{\varepsilon_{p},\theta ,T,\dot{\varepsilon }}=A-B*exp(-C*\varepsilon_{p})$	
**Loading state**	**Parameter**	
TTC	A	257.5
	B	145.1
	C	11.76
	R-squared	0.996
IPC	A	278.6
	B	248.2
	C	11.96
	R-squared	0.9645
**Sigmoidal-type hardening law**	$\sigma_{\varepsilon_{p},\theta ,T,\dot{\varepsilon }}=\frac{F_{1}-F_{4}}{1+exp(-\frac{\varepsilon_{p}-F_{2}}{F_{3}})}+F_{4}$	
**Loading state**	**Parameter**	
TTC	$F_{1}$	256.8
	$F_{2}$	−0.1614
	$F_{3}$	0.07881
	$F_{4}$	−1000
	R-squared	0.995
IPC	$F_{1}$	256.2
	$F_{2}$	0.05165
	$F_{3}$	0.03
	$F_{4}$	4.596
	R-squared	0.9938

**Table 2:** Strain rate hardening parameters of the flow curve parameters for Ta–10W (BCC).

$h^{\theta }(\dot{\varepsilon })=D_{1}^{\theta }\cdot \ln\left(\frac{\dot{\varepsilon }}{{\dot{\varepsilon }}_{0}}\right)+exp(D_{2}^{\theta }\cdot \frac{\dot{\varepsilon }}{{\dot{\varepsilon }}_{0}})$								
	$\frac{F_{1,\dot{\varepsilon }}}{F_{1,{\dot{\varepsilon }}_{0}}}$		$\frac{F_{2,\dot{\varepsilon }}}{F_{2,{\dot{\varepsilon }}_{0}}}$		$\frac{F_{3,\dot{\varepsilon }}}{F_{3,{\dot{\varepsilon }}_{0}}}$		$\frac{F_{4,\dot{\varepsilon }}}{F_{4,{\dot{\varepsilon }}_{0}}}$	
	$D_{1}^{\theta }$	$D_{2}^{\theta }$	$D_{1}^{\theta }$	$D_{2}^{\theta }$	$D_{1}^{\theta }$	$D_{2}^{\theta }$	$D_{1}^{\theta }$	$D_{2}^{\theta }$
Tension	0.007167	8.05 × 10^−15^	0.003739	−3.19 × 10^−16^	−0.0377	9.15 × 10^−15^	0.033	2.15 × 10^−14^
Compression	−0.0007421	−6.79 × 10^−16^	−0.5751	4.65 × 10^−22^	−0.07152	−8.19 × 10^−14^	1.976	5.68 × 10^−13^

**Table 3:** Temperature-related thermal softening parameters of the flow curve parameters for 2024-T351 (FCC).

$g^{\theta }\left(T\right)=C_{1}^{\theta }\cdot \exp\left(-C_{2}^{\theta }\cdot T\right)+C_{3}^{\theta }$												
	$\frac{F_{1,T}}{F_{1,T0}}$			$\frac{F_{2,T}}{F_{2,T0}}$			$\frac{F_{3,T}}{F_{3,T0}}$			$\frac{F_{4,T}}{F_{4,T0}}$		
	$C_{1}^{\theta }$	$C_{2}^{\theta }$	$C_{3}^{\theta }$	$C_{1}^{\theta }$	$C_{2}^{\theta }$	$C_{3}^{\theta }$	$C_{1}^{\theta }$	$C_{2}^{\theta }$	$C_{3}^{\theta }$	$C_{1}^{\theta }$	$C_{2}^{\theta }$	$C_{3}^{\theta }$
Tension	1.104	0.004705	0.6132	3.648	0.008585	0.4623	4.964	0.008372	0.2326	0.01144	−0.0076	0.9377
Compression	−0.009366	−0.007881	1.054	−8.75 × 10^−6^	−0.02585	1.003	−1.428 × 10^−4^	−0.01952	1.011	0.9888	0.00522	0.6914

**Table 4:** Temperature-related thermal softening parameters for AZ31’s flow curve parameters at a strain rate of $10^{-4}/s$.

$g^{\theta }\left(T\right)=C_{1}^{\theta }\cdot \exp\left(-C_{2}^{\theta }\cdot T\right)+C_{3}^{\theta }$													
		$\frac{F_{1,T}}{F_{1,T0}}$			$\frac{F_{2,T}}{F_{2,T0}}$			$\frac{F_{3,T}}{F_{3,T0}}$			$\frac{F_{4,T}}{F_{4,T0}}$		
		$C_{1}^{\theta }$	$C_{2}^{\theta }$	$C_{3}^{\theta }$	$C_{1}^{\theta }$	$C_{2}^{\theta }$	$C_{3}^{\theta }$	$C_{1}^{\theta }$	$C_{2}^{\theta }$	$C_{3}^{\theta }$	$C_{1}^{\theta }$	$C_{2}^{\theta }$	$C_{3}^{\theta }$
Tension	0°	3.847	0.003476	−0.3652	1 × 10^11^	0.08717	0.4773	−2.33 × 10^−7^	−0.03423	1.006	3.877	0.005205	0.1781
	45°	−8.416	−3.97 × 10^−4^	10.47	2.514	0.003206	0.0329	−1.28 × 10^−8^	−0.04078	1.002	−0.00011	−0.01944	1.036
	90°	−2.251	−0.00113	4.156	9.87 × 10^7^	0.06723	0.8037	−8.86 × 10^−7^	−0.03146	1.011	−5.27 × 10^−6^	−0.02604	1.012
Compression	0°	5.168	0.003007	−1.11	−1.29 × 10^−4^	−0.02162	1.081	5.43 × 10^−7^	−0.03153	0.994	−0.00752	−0.01191	1.261
	45°	5.585	0.00208	−2.008	−1 × 10^−4^	−0.02181	1.067	34.84	0.01908	0.882	−0.00509	−0.01146	1.155
	90°	−0.00408	−0.01259	1.174	−6.62 × 10^−10^	−0.05041	1.002	−15.63	0.01274	1.351	−0.00569	−0.01253	1.238

**Table 5:** Strain rate hardening parameters for AZ31’s flow curve parameters at 298 K.

$h^{\theta }(\dot{\varepsilon })=D_{1}^{\theta }\cdot \ln\left(\frac{\dot{\varepsilon }}{{\dot{\varepsilon }}_{0}}\right)+exp(D_{2}^{\theta }\cdot \frac{\dot{\varepsilon }}{{\dot{\varepsilon }}_{0}})$									
		$\frac{F_{1,\dot{\varepsilon }}}{F_{1,{\dot{\varepsilon }}_{0}}}$		$\frac{F_{2,\dot{\varepsilon }}}{F_{2,{\dot{\varepsilon }}_{0}}}$		$\frac{F_{3,\dot{\varepsilon }}}{F_{3,{\dot{\varepsilon }}_{0}}}$		$\frac{F_{4,\dot{\varepsilon }}}{F_{4,{\dot{\varepsilon }}_{0}}}$	
		$D_{1}^{\theta }$	$D_{2}^{\theta }$	$D_{1}^{\theta }$	$D_{2}^{\theta }$	$D_{1}^{\theta }$	$D_{2}^{\theta }$	$D_{1}^{\theta }$	$D_{2}^{\theta }$
Tension	0°	−0.004481	1.062 × 10^−5^	−0.05066	−3.284 × 10^−5^	−0.03407	5.17 × 10^−5^	0.01372	−4.585 × 10^−5^
	45°	0.01526	−5.79 × 10^−6^	−0.1208	2.345 × 10^−5^	0.1151	−2.151 × 10^−4^	−0.05519	2.707 × 10^−5^
	90°	0.01686	−9.918 × 10^−6^	−0.1709	3.102 × 10^−5^	0.07777	−1.207 × 10^−4^	−0.07312	3.413 × 10^−5^
Compression	0°	6.514 × 10−4	2.21 × 10^−5^	−0.01305	1.42 × 10^−5^	−0.03598	3.61 × 10^−5^	0.007793	−4.79 × 10^−6^
	45°	−0.02417	2.379 × 10^−5^	−0.02247	1.089 × 10^−5^	−0.06936	3.4 × 10^−5^	0.01629	−7.885 × 10^−6^
	90°	0.05245	−3.103 × 10^−5^	0.02037	−1.998 × 10^−5^	0.01319	−2.536 × 10^−5^	0.005495	−1.592 × 10^−6^

## Data Availability

The datasets used or analyzed during the current study are available from the corresponding author on reasonable request due to privacy.

## Appendix A

**Table A1:** Experimental data and texture simulation results of AZ31B [34].

Loading State	Experiment		Texture Simulation	
	Plastic Strain [–]	True Stress [MPa]	True Strain [-]	True Stress [MPa]
TTC	0	110	0	110
	0.025	150	0.025	150
	0.05	180	0.05	180
	0.075	200	0.075	200
	0.1	215	0.1	215
	0.125	225	0.125	225
	0.15	230	0.15	230
	0.175	235	0.175	235
	0.2	240	0.2	240
	0.225	245	0.225	245
	0.25	247.5	0.25	245
	0.275	250	0.275	250
	0.3	255	0.3	250
	0.325	255	0.325	250
	0.35	255	0.35	255
	0.375	260	0.375	255
	0.4	260	0.4	255
	-	-	0.425	255
	-	-	0.45	255
	-	-	0.475	255
	-	-	0.5	255
IPC	0	47.5	0	56.5
	0.025	75	0.025	75
	0.05	115	0.05	140
	0.075	187.5	0.075	190
	0.1	220	0.1	220
	0.125	237.5	0.125	235
	0.15	245	0.15	240
	0.175	247.5	0.175	245
	0.2	252.5	0.2	250
	0.225	255	0.225	255
	0.25	257.5	0.25	260
	-	-	0.275	262.5
	-	-	0.3	265
	-	-	0.325	267.5
	-	-	0.35	270
	-	-	0.375	270
	-	-	0.4	270
	-	-	0.425	270
	-	-	0.45	272.5
	-	-	0.475	272.5
	-	-	0.5	272.5

**Table A2:** Experimental data of Ta–10W (BCC) [35].

Strain Rate	Tension		Compression	
	True Strain [-]	True Stress [MPa]	True Strain [-]	True Stress [MPa]
0.001/s	0.002	500	0	458
	0.025	490	0.025	510
	0.05	540	0.05	560
	0.075	585	0.075	600
	0.1	620	0.1	640
	0.125	650	0.125	675
	0.15	675	0.15	700
	0.175	690	0.175	720
	0.2	700	0.2	740
1/s	0.002	590	0	556
	0.025	580	0.025	590
	0.05	610	0.05	630
	0.075	650	0.075	670
	0.1	680	0.1	710
	0.125	710	0.125	740
	0.15	730	0.15	760

**Table A3:** Experimental data of 2024-T351 (FCC) [36].

Temperature	Tension		Compression	
	True Strain [-]	True Stress [MPa]	True Strain [-]	True Stress [MPa]
−50 °C (223 K)	0.002	400	0	340
	0.02	420	0.02	400
	0.04	450	0.04	440
	0.06	485	0.06	490
	0.08	490	0.08	510
	0.1	510	0.1	530
	0.12	530	0.12	550
	0.14	550	0.14	570
	0.16	560	0.16	580
	0.18	580	0.18	590
	0.2	590	0.2	600
	0.22	590	0.22	610
	0.24	-	0.24	610
	0.26	-	0.26	620
	0.28	-	0.28	620
	0.3	-	0.3	630
	0.32	-	0.32	635
	0.34	-	0.34	935
	0.36	-	0.36	640
25 °C (298 K)	0.002	360	0	340
	0.02	400	0.02	400
	0.04	430	0.04	440
	0.06	460	0.06	480
	0.08	480	0.08	500
	0.1	500	0.1	510
	0.12	510	0.12	540
	0.14	530	0.14	550
	0.16	540	0.16	560
	0.18	550	0.18	570
	0.2	-	0.2	580
	0.22	-	0.22	585
	0.24	-	0.24	590
	0.26	-	0.26	595
	0.28	-	0.28	600
	0.3	-	0.3	605
	0.32	-	0.32	610
	0.34	-	0.34	610
150 °C (423 K)	0.002	330	0	300
	0.02	380	0.02	380
	0.04	410	0.04	425
	0.06	440	0.06	450
	0.08	460	0.08	480
	0.1	480	0.1	490
	0.12	480	0.12	490
	0.14	490	0.14	495
	0.16	490	0.16	495
	0.18	490	0.18	500
	0.2	-	0.2	505
	0.22	-	0.22	505
	0.24	-	0.24	505
	0.26	-	0.26	505
	0.28	-	0.28	510
	0.3	-	0.3	510
	0.32	-	0.32	510
	0.34	-	0.34	510

**Table A4:** Experimental data of AZ31 (HCP) [37].

Temperature	Strain Rate	Tension				Compression			
		True Strain	0°	45°	90°	True Strain	0°	45°	90°
25 °C (298 K)	0.0001/s	0.002	170.82	177.13	191.83	0.002	96.58	94.5	103.38
		0.0125	180	190	205	0.0075	97	97.5	105
		0.025	200	210	225	0.015	97.5	100	107.5
		0.0375	220	235	242.5	0.03	100	105	112.5
		0.05	235	250	255	0.045	115	120	132.5
		0.0625	250	260	265	0.06	140	145	155
		0.075	260	265	272.5	0.075	175	180	200
		0.0875	270	275	280	0.09	230	240	257.5
		0.1	275	280	285	0.1	250	260	270
	0.01/s	0.002	180	185	200	0.002	100	102	106
		0.0125	192.5	205	215	0.0075	101	101	107.5
		0.025	210	222.5	237.5	0.015	102.5	102.5	110
		0.0375	232.5	240	255	0.03	105	105	115
		0.05	250	260	270	0.045	115	115	125
		0.0625	257.5	267.5	277	0.06	145	147.5	155
		0.075	265	275	285	0.075	195	197.5	210
		0.0875	270	283.5	292.5	0.09	250	250	292.5
		0.1	275	287.5	300	0.1	267.5	267.5	302.5
	1/s	0.002	190	205	215	0.002	99	102	107
		0.0125	205	225	240	0.0075	100	103	108
		0.025	226	245	257.5	0.015	101	106	109
		0.0375	244	262.5	272	0.03	103	108	113
		0.05	255	272.5	282.5	0.045	123	123	131
		0.0625	265	283	292.5	0.06	150	152.5	172.5
		0.075	275	292	300	0.075	200	220	235
		0.1	287.5	300	305	0.09	277.5	285	315
		-	-	-	-	0.1	290	300	325
65 °C (338 K)	0.0001/s	0.002	157.18	160.93	179.18	0.002	81.18	86.39	87.36
		0.0125	170	177.5	192.5	0.0075	82.5	87	91
		0.025	187.5	192.5	205	0.015	85	87.5	94
		0.0375	200	205	215	0.03	90	96	100
		0.05	210	215	225	0.045	102.5	110	117.5
		0.0625	215	222.5	232.5	0.06	127.5	132.5	145
		0.075	225	230	237.5	0.075	157.5	162.5	175
		0.1	230	240	245	0.09	195	205	220
		-	-	-	-	0.1	200	215	240
	0.01/s	0.002	165	168	186	0.002	84	88	94
		0.0125	187.5	195	210	0.0075	90	92.5	97.5
		0.025	212.5	225	237.5	0.015	92.5	97.5	102.5
		0.0375	230	240	250	0.03	102.5	105	110
		0.05	245	255	260	0.045	117.5	120	125
		0.0625	253	257.5	265	0.06	147.5	152.5	152.5
		0.075	259	262.5	270	0.075	175	200	195
		0.1	262.5	267.5	275	0.09	225	242.5	250
		-	-	-	-	0.1	250	265	285
	1/s	0.002	180	190	210	0.002	98.5	101	106
		0.0125	200	217.5	235	0.0075	99	102	107
		0.025	225	242.5	252.5	0.015	100	105	110
		0.0375	242.5	260	270	0.03	102.5	107.5	112.5
		0.05	252.5	272.5	280	0.045	122.5	122.5	130
		0.0625	262.5	282.5	290	0.06	147.5	155	155
		0.075	272.5	290	297.5	0.075	190	210	225
		0.1	280	297.5	302.5	0.09	240	265	300
		-	-	-	-	0.1	267.5	292.5	330
150 °C (423 K)	0.0001/s	0.002	100	101.22	113.3	0.002	63	68.8	72.8
		0.0125	112.5	112.5	122.5	0.0075	72.5	77	80
		0.025	125	125	132.5	0.015	79	83	86
		0.0375	135	135	143	0.03	86	90.25	93
		0.05	143	143	148	0.04	90	94.25	97
		0.0625	145	145	150	0.045	91.5	95.75	98.5
		0.075	145	145	150.5	0.06	95	98.5	101
		0.1	145	145	151	0.07	97.5	100	102
	0.01/s	0.002	130	131	145	0.002	80	86	91
		0.0125	141	145	155	0.0075	84	87.5	92.5
		0.025	152.5	157.5	167.5	0.015	88	90	95
		0.0375	162.5	170	180	0.03	90	95	102.5
		0.05	172.5	180	188	0.045	105	112.5	130
		0.0625	180	186	195	0.06	130	137.5	150
		0.075	185	190	200	0.075	147.5	147.5	157.5
		0.1	190	195	205	0.09	152.5	152.5	159
		-	-	-	-	0.1	154	155	160
	1/s	0.002	170	180	195	0.002	98	100	105
		0.0125	180	190	205	0.0075	98.5	101	106
		0.025	195	205	217.5	0.015	100	102	107.5
		0.0375	205	217.5	227.5	0.03	107.5	105	110
		0.05	213	225	236	0.045	120	120	127.5
		0.0625	220	235	243	0.06	147.5	145	150
		0.075	225	240	247.5	0.075	170	170	175
		0.1	230	245	252.5	0.09	185	190	202.5
		-	-	-	-	0.1	190	192.5	205

## Appendix B

**Table A5:** Fitted model parameters for Ta-10W (BCC) with different hardening laws.

Swift Hardening Law	$\sigma_{\varepsilon_{p},\theta ,T,\dot{\varepsilon }}=A*{\left(\varepsilon_{p}+B\right)}^{n}$		
Strain Rate	Parameter	Tension	Compression
0.001/s	A	1149	1141
	B	0.1352	0.05008
	n	0.436	0.3073
	R-squared	0.9669	0.998
1/s	A	1000	1329
	B	0.6088	0.1368
	n	1.134	0.4415
	R-squared	0.9574	0.996
Ludwik hardening law	$\sigma_{\varepsilon_{p},\theta ,T,\dot{\varepsilon }}=\sigma_{y}+k*{\varepsilon_{p}}^{n}$		
Strain rate	Parameter	Tension	Compression
0.001/s	$\sigma_{y}$	484	453.5
	k	1014	933.2
	n	0.9116	0.7133
	R-squared	0.9617	0.9947
1/s	$\sigma_{y}$	569.3	552.9
	k	1051	1176
	n	0.99	0.8962
	R-squared	0.9554	0.9944
Voce hardening law	$\sigma_{\varepsilon_{p},\theta ,T,\dot{\varepsilon }}=A-B*exp(-C*\varepsilon_{p})$		
Strain rate	Parameter	Tension	Compression
0.001/s	A	972.1	871.3
	B	493.6	415.4
	C	3.185	5.829
	R-squared	0.969	0.9994
1/s	A	2000	1138
	B	1431	586
	C	0.7906	3.012
	R-squared	0.953	0.9965
Sigmoidal-type hardening law	$\sigma_{\varepsilon_{p},\theta ,T,\dot{\varepsilon }}=\frac{F_{1}-F_{4}}{1+\exp(-\frac{\varepsilon_{p}-F_{2}}{F_{3}})}+F_{4}$		
Strain rate	Parameter	Tension	Compression
0.001/s	$F_{1}$	702.9	799.8
	$F_{2}$	0.08013	−0.01945
	$F_{3}$	0.03348	0.08932
	$F_{4}$	469.8	33.42
	R-squared	0.99	0.9998
1/s	$F_{1}$	737.7	795.7
	$F_{2}$	0.0834	0.05782
	$F_{3}$	0.02476	0.04519
	$F_{4}$	576.9	489.7
	R-squared	0.9887	0.9999

**Table A6:** Fitted model parameters for 2024-T351 (FCC) with different hardening laws.

Swift Hardening Law	$\sigma_{\varepsilon_{p},\theta ,T,\dot{\varepsilon }}=A*{\left(\varepsilon_{p}+B\right)}^{n}$		
Temperature	Parameter	Tension	Compression
−50 °C (223 K)	A	851.9	776.2
	B	0.06606	0.007767
	n	0.2819	0.1732
	R-squared	0.9926	0.9882
25 °C (298 K)	A	779.7	737.8
	B	0.02961	0.007655
	n	0.2206	0.1616
	R-squared	0.9985	0.991
150 °C (423 K)	A	630.9	576.5
	B	0.008222	0.0006734
	n	0.1363	0.08983
	R-squared	0.9791	0.9599
Ludwik hardening law	$\sigma_{\varepsilon_{p},\theta ,T,\dot{\varepsilon }}=\sigma_{y}+k*{\varepsilon_{p}}^{n}$		
Temperature	Parameter	Tension	Compression
−50 °C (223 K)	$\sigma_{y}$	394.8	328.9
	k	624.1	496.8
	n	0.7286	0.4047
	R-squared	0.9891	0.9748
25 °C (298 K)	$\sigma_{y}$	357.3	331.7
	k	583.3	455.6
	n	0.6295	0.4037
	R-squared	0.995	0.9791
150 °C (423 K)	$\sigma_{y}$	326.8	296.5
	k	381.9	292.7
	n	0.4454	0.2321
	R-squared	0.9668	0.9459
Voce hardening law	$\sigma_{\varepsilon_{p},\theta ,T,\dot{\varepsilon }}=A-B*exp(-C*\varepsilon_{p})$		
Temperature	Parameter	Tension	Compression
−50 °C (223 K)	A	697.4	643.3
	B	300.2	299
	C	4.953	9.929
	R-squared	0.9935	0.9975
25 °C (298 K)	A	598.7	614.8
	B	237.8	268.4
	C	8.708	10.35
	R-squared	0.9989	0.9968
150 °C (423 K)	A	502.2	506.7
	B	173.7	204.2
	C	17.43	22.35
	R-squared	0.995	0.9957
Sigmoidal-type hardening law	$\sigma_{\varepsilon_{p},\theta ,T,\dot{\varepsilon }}=\frac{F_{1}-F_{4}}{1+\exp(-\frac{\varepsilon_{p}-F_{2}}{F_{3}})}+F_{4}$		
Temperature	Parameter	Tension	Compression
−50 °C (223 K)	$F_{1}$	654.4	639.4
	$F_{2}$	−0.05445	−0.1348
	$F_{3}$	0.115	0.08828
	$F_{4}$	−5.726	−1000
	R-squared	0.9937	0.9977
25 °C (298 K)	$F_{1}$	575.6	611.4
	$F_{2}$	−0.04055	−0.1326
	$F_{3}$	0.07385	0.08505
	$F_{4}$	−6	−900
	R-squared	0.9981	0.9956
150 °C (423 K)	$F_{1}$	497	506.1
	$F_{2}$	−0.03043	−0.06894
	$F_{3}$	0.04329	0.04063
	$F_{4}$	−7	−800
	R-squared	0.9966	0.9946

**Table A7:** Fitted model parameters for AZ31 (HCP) with different hardening laws.

Swift Hardening Law		$\sigma_{\varepsilon_{p},\theta ,T,\dot{\varepsilon }}=A*{\left(\varepsilon_{p}+B\right)}^{n}$						
Temperature	Strain Rate	Parameters	Tension			Compression		
			0°	45°	90°	0°	45°	90°
25 °C (298 K)	0.0001/s	A	598.2	501.4	475.5	6.69 × 10^−8^	1.049 × 10^−7^	5.1 × 10^−8^
		B	0.03327	0.01807	0.02006	2.43	2.319	2.426
		n	0.375	0.2643	0.2353	23.68	24.44	24.13
		R-squared	0.9923	0.9863	0.9947	0.9292	0.9496	0.9475
	0.01/s	A	476.8	485	485.4	8.75 × 10^−8^	1.156 × 10^−7^	3.88 × 10^−9^
		B	0.01826	0.01604	0.01708	2.224	2.282	2.482
		n	0.2482	0.2351	0.2206	25.91	24.81	26.38
		R-squared	0.9831	0.993	0.9944	0.9472	0.9392	0.9086
	1/s	A	482.1	453.7	425.9	1.179 × 10^−7^	4.654 × 10^−8^	4.05 × 10^−8^
		B	0.01891	0.01244	0.008391	2.193	2.33	2.292
		n	0.2372	0.1825	0.1435	26.03	25.38	26.11
		R-squared	0.9958	0.9953	0.9953	0.9387	0.9174	0.9264
65 °C (338 K)	0.0001/s	A	356.7	376.5	359.2	5.823 × 10^−8^	6.31 × 10^−8^	5.83 × 10^−8^
		B	0.01398	0.01551	0.01803	2.685	2.793	2.57
		n	0.1942	0.2047	0.1741	21.38	20.57	22.46
		R-squared	0.9916	0.8768	0.9272	0.9091	0.9948	0.9908
	0.01/s	A	408.5	388.6	377.9	3.513 × 10^−8^	4.33 × 10^−8^	7.44 × 10^−8^
		B	0.00673	0.003875	0.004375	2.437	2.428	2.366
		n	0.1837	0.1526	0.1318	24.3	24.23	24.35
		R-squared	0.9826	0.9789	0.9849	0.9552	0.9423	0.9398
	1/s	A	439.7	445.7	430.1	5.901 × 10^−8^	5.70 × 10^−8^	7.41 × 10^−8^
		B	0.01004	0.006857	0.008367	2.362	2.282	2.206
		n	0.1962	0.1724	0.1506	24.61	25.71	26.53
		R-squared	0.9928	0.9942	0.9936	0.9473	0.946	0.9305
150 °C (423 K)	0.0001/s	A	202.7	203.1	200.1	141.5	141.6	142.3
		B	0.00416	0.004687	0.005737	0.003416	0.003852	0.004298
		n	0.1306	0.1318	0.1119	0.1423	0.1301	0.1234
		R-squared	0.9554	0.9526	0.9558	0.9996	0.9987	0.9971
	0.01/s	A	307	298.8	315.9	1166	763.1	469.4
		B	0.01771	0.01104	0.0166	0.1419	0.09342	0.03687
		n	0.2152	0.1848	0.1925	1.39	0.943	0.5154
		R-squared	0.9913	0.9896	0.9895	0.9598	0.9594	0.9449
	1/s	A	331.6	365.9	352.2	8.57 × 10^−9^	4.795 × 10^−7^	2.81 × 10^−7^
		B	0.01559	0.01822	0.01852	3.034	2.543	2.578
		n	0.1626	0.1796	0.1498	20.86	20.43	20.75
		R-squared	0.9914	0.9886	0.9921	0.962	0.968	0.9711
Ludwik hardening law		$\sigma_{\varepsilon_{p},\theta ,T,\dot{\varepsilon }}=\sigma_{y}+k*{\varepsilon_{p}}^{n}$						
Temperature	Strain rate	Parameters	Tension			Compression		
			0°	45°	90°	0°	45°	90°
25 °C (298 K)	0.0001/s	$\sigma_{y}$	167.1	173.5	189.3	73.77	73.89	81.27
		k	708.7	535.5	475.9	1525	1621	1704
		n	0.7871	0.673	0.6715	0.99	0.99	0.99
		R-squared	0.9874	0.9774	0.9879	0.8815	0.894	0.9061
	0.01/s	$\sigma_{y}$	176.5	182.9	197.5	74.71	75.44	76.52
		k	493.7	482.7	473.3	1696	1697	1998
		n	0.6672	0.6371	0.6457	0.99	0.99	0.99
		R-Squared	0.9733	0.987	0.9875	0.8747	0.8767	0.86
	1/s	$\sigma_{y}$	187.7	203.1	213.8	69.93	72.1	73.53
		k	486.5	400.7	325.8	1949	2036	2282
		n	0.6642	0.588	0.5225	0.99	0.99	0.99
		R-squared	0.9892	0.9874	0.9885	0.873	0.8748	0.8844
65 °C (338 K)	0.0001/s	$\sigma_{y}$	155.4	159.8	178	66.34	69.6	70.85
		k	372.3	350.8	302.4	1254	1330	1513
		n	0.61	0.6214	0.6292	0.99	0.99	0.99
		R-squared	0.9822	0.9948	0.9908	0.9279	0.9252	0.9313
	0.01/s	$\sigma_{y}$	162.5	165.9	184.3	68.23	69.18	72.38
		k	362.1	308	274.6	1603	1772	1819
		n	0.5161	0.4438	0.4473	0.99	0.99	0.99
		R-squared	0.969	0.9643	0.9712	0.9316	0.93	0.905
	1/s	$\sigma_{y}$	177.8	188.1	208.6	74.42	73.69	72.97
		k	407.2	382.9	339.2	1670	1908	2254
		n	0.57	0.514	0.5252	0.99	0.99	0.99
		R-squared	0.9833	0.9851	0.9855	0.8955	0.888	0.8641
150 °C (423 K)	0.0001/s	$\sigma_{y}$	98.75	99.88	112.1	62.79	68.44	72.35
		k	144.1	145.3	128.2	126.7	119.5	116.2
		n	0.4358	0.4486	0.4624	0.477	0.4813	0.4895
		R-squared	0.9358	0.9319	0.9344	0.9982	0.9937	0.99
	0.01/s	$\sigma_{y}$	128.6	129.5	143.3	76.33	81.88	86.14
		k	293.2	265.7	286.6	844	800.7	613.8
		n	0.6443	0.5735	0.6314	0.99	0.99	0.8655
		R-squared	0.9832	0.9794	0.9791	0.9588	0.9594	0.9395
	1/s	$\sigma_{y}$	168.3	178	193.5	88.18	88.74	92.75
		k	273	318.2	271.4	1037	1047	1113
		n	0.6127	0.6398	0.6288	0.99	0.99	0.99
		R-squared	0.9808	0.978	0.9823	0.9621	0.9462	0.9443
Voce hardening law		$\sigma_{\varepsilon_{p},\theta ,T,\dot{\varepsilon }}=A-B*exp(-C*\varepsilon_{p})$						
Temperature	Strain rate	Parameters	Tension			Compression		
			0°	45°	90°	0°	45°	90°
25 °C (298 K)	0.0001/s	A	347.7	310	314	1074	973.8	1281
		B	180.7	136.7	124.3	1000	900	1200
		C	9.759	15.91	15.13	1.66	1.987	1.543
		R-squared	0.9946	0.9918	0.9979	0.8699	0.8804	0.8969
	0.01/s	A	302.1	314.6	324.1	1075	1175	1227
		B	126.1	130.7	125.9	1000	1100	1150
		C	16.45	16.56	16.52	1.86	1.682	1.907
		R-squared	0.9901	0.9966	0.9972	0.8612	0.865	0.846
	1/s	A	316.6	317	315.5	1170	1272	1373
		B	128	112.5	99.61	1100	1200	1300
		C	15.24	19.81	23.89	1.949	1.861	1.933
		R-squared	0.9984	0.9992	0.9982	0.859	0.8617	0.8714
65 °C (338 K)	0.0001/s	A	245	258.6	261.6	1066	1169	1221
		B	89.03	97.32	82.6	1000	1100	1150
		C	18.95	16.71	16.72	1.356	1.303	1.42
		R-squared	0.9966	0.9998	0.9993	0.905	0.91	0.92
	0.01/s	A	273.2	271.7	278.2	1068	1169	1271
		B	110.1	105.2	93.09	1000	1100	1200
		C	26.75	34.22	33.64	1.76	1.769	1.653
		R-squared	0.9967	0.9967	0.9978	0.9218	0.8867	0.8944
	1/s	A	294.6	307.4	313.4	1274	1324	1373
		B	115.8	117.5	103	1200	1250	1300
		C	21.62	25.59	24.12	1.509	1.664	1.859
		R-squared	0.9982	0.9999	0.9984	0.8856	0.8767	0.848
150 °C (423 K)	0.0001/s	A	149	149.2	154.5	98.99	101.8	104
		B	50.36	49.59	42.61	34.55	32.1	30.66
		C	35.76	34.68	33.41	37.7	39.34	39.34
		R-squared	0.9867	0.9844	0.9847	0.9907	0.9963	0.9979
	0.01/s	A	206.9	205.4	219.4	1000	833.1	229.1
		B	77.9	75.26	75.95	924.1	751.9	144.2
		C	16.59	21.43	18.01	0.9571	1.13	8.403
		R-squared	0.9965	0.9973	0.9962	0.958	0.9596	0.9492
	1/s	A	242.4	261.8	266.4	1188	1238	1292
		B	73.74	83.59	72.51	1100	1150	1200
		C	19.09	17.55	17.84	0.987	0.9504	0.9684
		R-squared	0.9974	0.9949	0.9974	0.9588	0.9421	0.9395
Sigmoidal-type hardening law		$\sigma_{\varepsilon_{p},\theta ,T,\dot{\varepsilon }}=\frac{F_{1}-F_{4}}{1+\exp(-\frac{\varepsilon_{p}-F_{2}}{F_{3}})}+F_{4}$						
Temperature	Strain rate	Parameters	Tension			Compression		
			0°	45°	90°	0°	45°	90°
25 °C (298 K)	0.0001/s	$F_{1}$	281	281.3	291	287.8	319.5	307.4
		$F_{2}$	0.03242	0.02242	0.02081	0.07702	0.07971	0.0352
		$F_{3}$	0.0241	0.02121	0.026	0.0145	0.01693	0.01512
		$F_{4}$	145	140	150	96.58	94.95	103.4
		R-squared	0.9986	0.9961	0.9977	0.9982	0.9976	0.997
	0.01/s	$F_{1}$	275.5	300.9	313.3	289.3	284.7	380.7
		$F_{2}$	0.02475	0.01	0.0045	0.0725	0.07155	0.08027
		$F_{3}$	0.01901	0.032	0.05	0.01215	0.01158	0.016
		$F_{4}$	153.5	104.8	100	100	102	106
		R-squared	0.998	0.9963	0.9954	0.9997	0.9996	0.9912
	1/s	$F_{1}$	295.5	305	308.7	360.7	334.2	373.9
		$F_{2}$	0.00822	0.0034	−0.00437	0.07949	0.07293	0.074
		$F_{3}$	0.03055	0.02495	0.0264	0.016	0.01297	0.01357
		$F_{4}$	110	113	110	99	102	107
		R-squared	0.998	0.9922	0.9962	0.9933	0.9981	0.9963
65 °C (338 K)	0.0001/s	$F_{1}$	231.2	238.7	248.4	219	242.9	272.4
		$F_{2}$	0.016	0.01981	0.017	0.06841	0.0229	0.0725
		$F_{3}$	0.022	0.021	0.02532	0.01474	0.01586	0.01724
		$F_{4}$	122.6	134.1	146.6	81.18	86.39	87.36
		R-squared	0.9955	0.9911	0.9971	0.9976	0.9973	0.9978
	0.01/s	$F_{1}$	265.2	272.6	280.6	272.5	268.1	312.5
		$F_{2}$	0.00402	0.0025	0.001	0.0705	0.06651	0.073
		$F_{3}$	0.02134	0.022	0.0225	0.0155	0.01153	0.015
		$F_{4}$	82.11	82.87	95	84	88	94
		R-squared	0.9997	0.9896	0.985	0.9905	0.99	0.9946
	1/s	$F_{1}$	282.9	303.1	306.5	309.5	315	345
		$F_{2}$	0.00559	0.00685	−0.0056	0.07685	0.07221	0.07238
		$F_{3}$	0.0247	0.02498	0.02643	0.01537	0.01239	0.01093
		$F_{4}$	100	115	95	98.5	101	106
		R-squared	0.997	0.9934	0.9975	0.9989	0.9996	0.9976
150 °C (423 K)	0.0001/s	$F_{1}$	145.8	145.9	151.2	97.59	99.79	102.9
		$F_{2}$	0.01547	0.01526	0.01673	−0.01018	0.00366	−0.01509
		$F_{3}$	0.01242	0.01287	0.01242	0.0929	0.01511	0.01935
		$F_{4}$	88	88.04	103.8	10	48	10
		R-squared	0.9969	0.9975	0.9999	0.985	0.988	0.996
	0.01/s	$F_{1}$	195.4	93.3	203.1	161.5	154.4	1159.5
		$F_{2}$	0.003	0.023	0.00098	0.05312	0.04738	0.04101
		$F_{3}$	0.03	0.021	0.0225	0.016	0.01051	0.00881
		$F_{4}$	70.2	71.87	83.24	80	86	91
		R-squared	0.9991	0.9923	0.9875	0.9905	0.9988	0.9993
	1/s	$F_{1}$	229.5	248.9	259.7	193.3	196.9	207.3
		$F_{2}$	−0.00234	0.01364	−0.01663	0.05773	0.06009	0.06097
		$F_{3}$	0.024	0.02501	0.03359	0.01279	0.012	0.0109
		$F_{4}$	98	139.6	90	98	100	105
		R-squared	0.992	0.9985	0.9992	0.9993	0.999	0.9931
